# *Poaceae*‐specific β‐1,3;1,4‐d‐glucans link jasmonate signalling to OsLecRK1‐mediated defence response during rice‐brown planthopper interactions

**DOI:** 10.1111/pbi.14038

**Published:** 2023-03-23

**Authors:** Yang‐Shuo Dai, Di Liu, Wuxiu Guo, Zhi‐Xuan Liu, Xue Zhang, Li‐Li Shi, De‐Mian Zhou, Ling‐Na Wang, Kui Kang, Feng‐Zhu Wang, Shan‐Shan Zhao, Yi‐Fang Tan, Tian Hu, Wu Chen, Peng Li, Qing‐Ming Zhou, Long‐Yu Yuan, Zhenfei Zhang, Yue‐Qin Chen, Wen‐Qing Zhang, Juan Li, Lu‐Jun Yu, Shi Xiao

**Affiliations:** ^1^ State Key Laboratory of Biocontrol, Guangdong Provincial Key Laboratory of Plant Resources, School of Life Sciences Sun Yat‐sen University Guangzhou China; ^2^ College of Agronomy Hunan Agricultural University Changsha China; ^3^ Plant Protection Research Institute Guangdong Academy of Agricultural Sciences Guangzhou China

**Keywords:** jasmonates, mixed‐linkage β‐1,3;1,4‐d‐glucan, OsLecRK1, OsCslF6, *Oryza sativa*, plant‐pest interaction

## Abstract

Brown planthopper (BPH, *Nilaparvata lugens*), a highly destructive insect pest, poses a serious threat to rice (*Oryza sativa*) production worldwide. Jasmonates are key phytohormones that regulate plant defences against BPH; however, the molecular link between jasmonates and BPH responses in rice remains largely unknown. Here, we discovered a Poaceae‐specific metabolite, mixed‐linkage β‐1,3;1,4‐d‐glucan (MLG), which contributes to jasmonate‐mediated BPH resistance. MLG levels in rice significantly increased upon BPH attack. Overexpressing *OsCslF6*, which encodes a glucan synthase that catalyses MLG biosynthesis, significantly enhanced BPH resistance and cell wall thickness in vascular bundles, whereas knockout of *OsCslF6* reduced BPH resistance and vascular wall thickness. OsMYC2, a master transcription factor of jasmonate signalling, directly controlled the upregulation of *OsCslF6* in response to BPH feeding. The AT‐rich domain of the *OsCslF6* promoter varies in rice varieties from different locations and natural variants in this domain were associated with BPH resistance. MLG‐derived oligosaccharides bound to the plasma membrane‐anchored LECTIN RECEPTOR KINASE1 OsLecRK1 and modulated its activity. Thus, our findings suggest that the OsMYC2‐OsCslF6 module regulates pest resistance by modulating MLG production to enhance vascular wall thickness and OsLecRK1‐mediated defence signalling during rice‐BPH interactions.

## Introduction

Jasmonates are a family of lipid‐derived signalling molecules, including jasmonic acid (JA), methyl jasmonate (MeJA), jasmonoyl‐L‐isoleucine (JA‐Ile), and 12‐oxophytodienoic acid (OPDA), that activate plant immunity against insect herbivores and pathogens (Howe *et al*., 2018; Wang *et al*., 2019). The molecular mechanisms for jasmonate perception (Fonseca *et al*., 2009; Hu *et al*., 2023; Katsir *et al*., 2008; Sheard *et al*., 2010; Thines *et al*., 2007; Yan *et al*., 2009, 2016, 2018) and signalling (An *et al*., 2017; Chen *et al*., 2012; Chini *et al*., 2007; Han, 2017; Howe *et al*., 2018; Kidd *et al*., 2009; Pauwels *et al*., 2010; Thines *et al*., 2007; Wan and Xin, 2022; Wang *et al*., 2019; Xie *et al*., 1998; Xu *et al*., 2002; Yan *et al*., 2007; Zhang *et al*., 2015) have been uncovered in model dicot plants. In *Arabidopsis thaliana*, the CORONATINE INSENSITIVE1 (COI1) and JASMONATE ZIM DOMAIN (JAZ) coreceptors perceive JA‐Ile, the most bioactive form of JA in plants, to initiate signalling (Fonseca *et al*., 2009; Sheard *et al*., 2010; Thines *et al*., 2007; Yan *et al*., 2009, 2016, 2018). In the absence of JA‐Ile, JAZs suppress the activities of defence‐associated transcription factors such as the basic helix–loop–helix (bHLH) proteins MYC2/3/4/5 and bHLH3/13/14/17, and a WD‐repeat/bHLH/MYB complex (Chini *et al*., 2007, 2016; Pauwels *et al*., 2010; Thines *et al*., 2007; Wang *et al*., 2019; Yan *et al*., 2007; Zhang *et al*., 2015). In response to insect feeding or pathogen infection, JA‐Ile accumulates and promotes the association of the E3 ubiquitin ligase SCF^COI1^with JAZ proteins, leading to their ubiquitination and degradation (Chini *et al*., 2007; Han, 2017; Howe *et al*., 2018; Thines *et al*., 2007; Wang *et al*., 2019; Xie *et al*., 1998; Xu *et al*., 2002; Zhang *et al*., 2015). The released MYC2 interacts with MED25, a subunit of the Mediator transcriptional co‐activator complex, to activate the expression of JA‐responsive defence genes (An *et al*., 2017; Chen *et al*., 2012; Kidd *et al*., 2009; Zhai *et al*., 2020).

Glucosinolates (GSs) are a class of secondary metabolites containing nitrogen and sulfur that are characteristic of the Brassicaceae family (Schweizer *et al*., 2013; Wang *et al*., 2019). Increasing evidence suggests that upon herbivore attack, a JAZ–MYC module regulates the expression of key GS biosynthesis genes to control the accumulation of these toxic compounds (Schweizer *et al*., 2013; Wang *et al*., 2019). In Arabidopsis, the *myc2/3/4* triple knockout mutant shows increased susceptibility to the feeding of generalist herbivores, suggesting that these MYC transcription factors redundantly modulate defences against insects in Brassicaceae plants (Fernandez‐Calvo *et al*., 2011; Major *et al*., 2017). Biochemical analyses revealed that the MYC2/3/4 transcription factors regulate GS biosynthesis by directly associating with the promoters of several GS biosynthesis genes or interacting with MYB factors involved in regulating GS biosynthesis (Liao *et al*., 2020; Schweizer *et al*., 2013). However, how JAs help regulate defence responses against pests in the Poaceae family remains largely unknown.

Brown planthopper (BPH, *Nilaparvata lugens*) is one of the most destructive pests of rice (*Oryza sativa*). BPH severely damages rice by sucking phloem sap from the leaf sheath, thus causing wilting and leading to yield losses (Cheng *et al*., 2013b). Studies of the rice‐BPH interaction have identified several BPH‐associated resistance genes (Du *et al*., 2009; Guo *et al*., 2018; Liu *et al*., 2015; Ren *et al*., 2016; Tamura *et al*., 2014; Wang *et al*., 2015; Zhao *et al*., 2016). For example, *Bph3* is a cluster of three genes encoding plasma membrane‐anchored lectin receptor kinases (OsLecRKs), which may act as sensors for herbivore‐associated molecular patterns (HAMPs) or damage‐associated molecular patterns (DAMPs) and activate plant defences (Liu *et al*., 2015; Reymond, 2021).

Some BPH resistance genes, such as *Bph6*, *Bph14*, and *Bph29*, produce broad plant resistance to BPH by modulating phytohormone signal transduction pathways (Du *et al*., 2009; Guo *et al*., 2018; Wang *et al*., 2015). Indeed, phytohormones including JA, salicylic acid (SA), and brassinosteroids play important roles in regulating plant responses to BPH feeding (Pan *et al*., 2018; Xu *et al*., 2021). For example, the R2R3 MYB transcription factor OsMYB30 regulates the expression of the phenylalanine ammonia‐lyase genes *OsPAL6* and *OsPAL8* to control BPH resistance and SA accumulation (He *et al*., 2020), providing a functional OsMYB30‐OsPAL‐SA module in rice BPH resistance.

Here, we report that mixed‐linkage β‐1,3;1,4‐d‐glucan (MLG) is a Poaceae‐specific metabolite that acts downstream of JA signalling to regulate BPH resistance. Overproduction of MLG enhances BPH resistance and causes thickening of the vascular bundles. Moreover, MLG‐derived oligosaccharides bind with high affinities to OsLecRK1 *in vitro* and modulate its activity *in vivo*, suggesting that MLGs may contribute to BPH resistance by enhancing vascular thickening and OsLecRK1‐mediated defence signalling during the rice‐BPH interaction.

## Results

### Mixed‐linkage β‐1,3;1,4‐d‐glucan is required for JA‐mediated BPH resistance

To investigate the potential role of JAs in defence against BPH in rice, we measured endogenous JA levels in rice at various time points (0, 6, 12, and 24 h) during BPH infestation. As shown in Figure S1a, compared to the untreated controls (0 h), the levels of OPDA, JA, and JA‐Ile in the leaf sheaths and stems of BPH‐infested plants were elevated beginning at 6 h of BPH infestation, with a peak at 12 h after treatment. Reverse transcription quantitative PCR (RT‐qPCR) analysis showed that the JA biosynthesis‐related gene *OsLOX2* and the JA signalling‐related genes *OsCOI1a* and *OsCOI1b* were significantly upregulated at 6, 12, and 24 h after BPH application (Figure S1b).

To further explore the roles of JAs in BPH resistance, we used CRISPR‐Cas9 to target the third exon of *OsLOX2*, the fourth exon of *OsCOI1a*, and the third exon of *OsCOI1b*. We generated two independent *oslox2* lines (designated *oslox2‐1* and *oslox2‐2*) and two *oscoi1a oscoi1b* double mutants (designated *oscoi1‐1* and *oscoi1‐2*) with knockout mutations of *OsLOX2* or *OsCOI1a* and *OsCOI1b*, respectively (Figure S2a,b). Compared to wild‐type Nipponbare (NIP), *oslox2‐1* and *oslox2‐2* plants had significantly reduced levels of wounding‐induced JA and JA‐Ile (Figure S2c). Exogenous application of MeJA at 5 and 10 μm significantly inhibited the root growth of NIP seedlings, whereas the *oscoi1* mutants were less sensitive to MeJA‐induced root inhibition than NIP (Figure S2d,e).

We next examined the responses of NIP, *oslox2* (*oslox2‐1* and *oslox2‐2*), and *oscoi1a/1b* (*oscoi1‐1* and *oscoi1‐2*) plants to BPH feeding. Four‐week‐old *oslox2* and *oscoi1* plants exhibited few morphological differences from NIP seedlings (Figure S3a). However, compared to NIP plants, *oslox2* and *oscoi1* plants were hypersensitive to BPH infestation (Figure S3a). The increased susceptibility of *oslox2* and *oscoi1* to BPH was further confirmed by measuring the survival rates of plants and the weight of honeydew excreted by BPH. Upon BPH feeding for 5 days, only 27% of *oslox2‐1* and *oslox2‐2* plants and 20% of *oscoi1‐1* and *oscoi1‐2* plants, survived compared to the 65% survival rate of NIP plants (Figure S3b). Consistent with these results, the honeydew weights were significantly higher on *oslox2* and *oscoi1* plants than on NIP (Figure S3b). When we sprayed NIP, *oslox2*, and *oscoi1* plants with 100 μm MeJA prior to BPH infestation, this treatment significantly reduced the susceptibility of *oslox2*, but not *oscoi1*, to BPH feeding (Figure S3c,d).

To identify potential JA‐regulated metabolites that change in abundance in response to BPH feeding, we performed liquid chromatography mass spectrometry (LC–MS)‐based widely targeted and untargeted metabolomic profiling of NIP, *oslox2*, and *oscoi1* plants that had undergone BPH treatment for 0 and 24 h. We used leaf sheath and stem samples because they are the major sites of BPH feeding. Our widely targeted metabolomics analysis identified 805 metabolites that matched known biochemicals (Dataset S1). Among these, the levels of 109 and 88 metabolites were significantly different in the NIP vs. *oslox2‐1* and NIP vs. *oscoi1‐1* groups, respectively (Figure S4a and Dataset S1). The levels of 24 metabolites were significantly different in NIP upon BPH exposure compared to NIP without treatment (Figure S4b; Dataset S1). In particular, the levels of four BPH‐inducible flavonoids, including one flavonoid (tricin‐4′‐methylether‐3′‐*O*‐glucoside), two flavonols (kaempferol‐4′‐*O*‐glucoside and isorhamnetin‐3,7‐*O*‐diglucoside), and one dihydroflavonol (dihydrokaempferol) were significantly lower in both *oslox2* and *oscoi1* compared to NIP (Figure S4c–e and Dataset S1).

**Figure 1:**
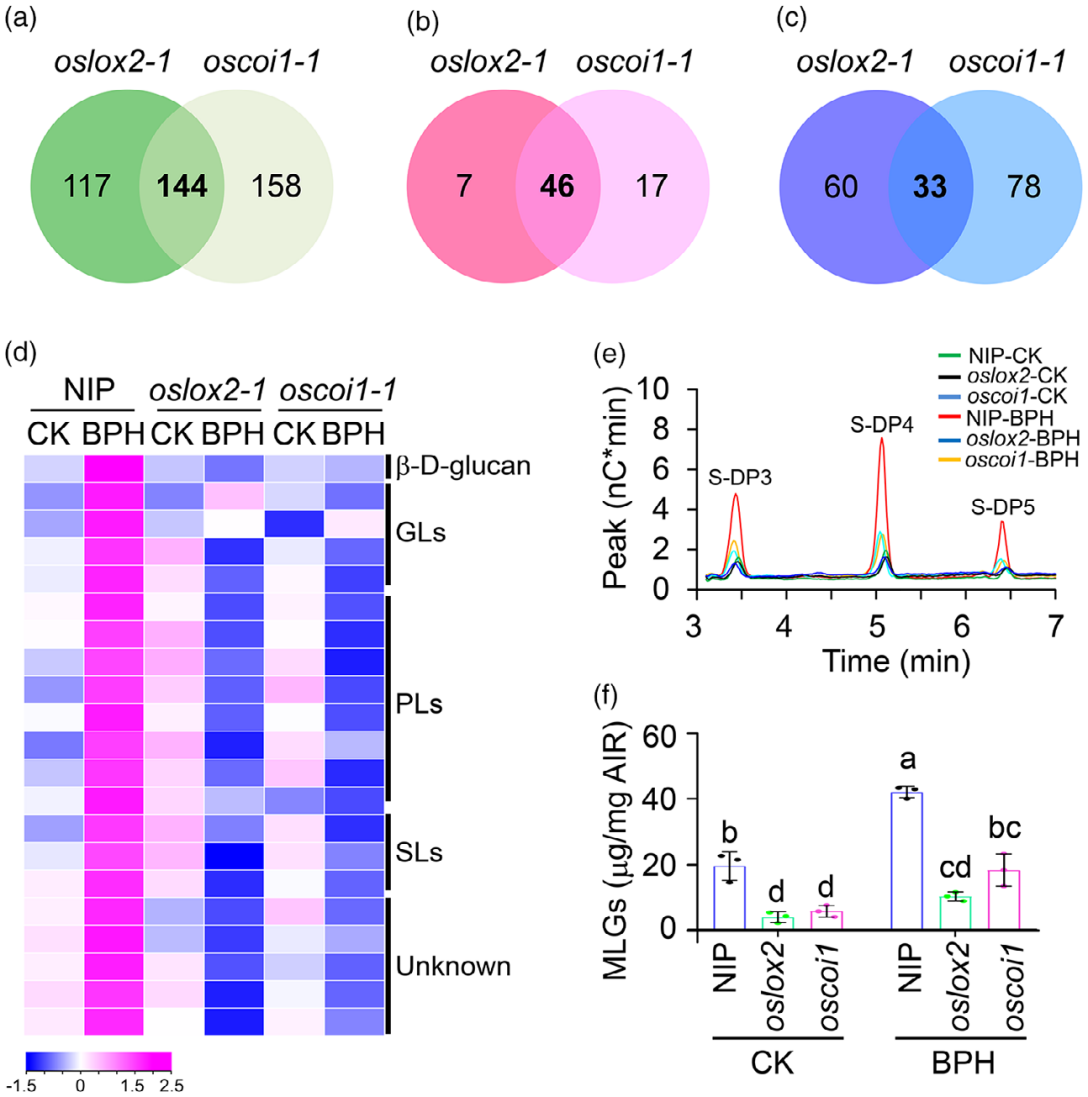
MLG is an essential metabolite for jasmonate‐mediated BPH resistance in rice. (a–c) Venn diagrams showing untargeted metabolomic profiling of plants upon BPH treatment. Leaf sheaths and stems of 4‐week‐old NIP, *oslox2‐1*, and *oscoi1‐1* plants at 0 (CK) and 1 days (BPH) after infestation with BPH were collected and subjected to untargeted metabolomic analysis, followed by analysis using XCMS Online software. The diagrams show the number of different features identified by one‐way ANOVA (*P* < 0.01) in each group of NIP vs. *oslox2‐1* (261) or NIP vs. *oscoi1‐1* (302) (a) and features that accumulated to significantly higher (b) or lower (c) levels in NIP plants compared to *oslox2‐1* and *oscoi1‐1* plants upon BPH infestation (*P* < 0.01). (d) Untargeted metabolomic profiling showing potential metabolites that accumulated upon BPH infestation in 4‐week‐old NIP plants but whose levels were significantly lower in the *oslox2‐1* and *oscoi1‐1* mutants (*P* < 0.01 by one‐way ANOVA). Standard‐scores (*Z*‐scores) were used to indicate the means of arbitrary peak abundance units (*n* = 4 biological replicates) of the corresponding samples. The first metabolite is β‐d‐glucan. (e) HPAEC analysis showing MLG levels in the leaf sheaths and stems of 4‐week‐old NIP, *oslox2*, and *oscoi1* plants upon BPH infestation for 0 (CK) and 1 day (BPH). Three biological replicates were conducted with similar results, and representative data from one replicate are shown. (f) MLG levels in the leaf sheaths and stems of 4‐week‐old NIP, *oslox2*, and *oscoi1* plants upon BPH infestation for 0 (CK) and 1 day (BPH). Data are means ± SD (*n* = 3 biological replicates). Letters indicate significant differences between groups conducted by one‐way ANOVA, *P* < 0.05.

We then performed untargeted metabolome profiling to identify possible Poaceae‐specific metabolites that might be affected during the BPH‐rice interaction. We detected approximately 800 features in the leaf sheaths and stems of rice plants (Dataset S2), including 261 and 302 features with significantly different levels in the NIP vs. *oslox2‐1* and NIP vs. *oscoi1‐1* groups, respectively (Figure 1a and Dataset S2). Upon BPH exposure, 46 features accumulated to significantly higher levels (compared with untreated plants) in NIP, but their levels were significantly lower in *oslox2* and *oscoi1* compared to NIP (Figure 1b,c and Dataset S2). By searching the METLIN database, we identified 16 of the 46 features, including one known metabolite (β‐d‐glucan), four glycerolipids (36:5‐, 36:7‐, 38:8‐MGDG, and 36:6 DGDG), eight phospholipids (34:2‐, 34:4‐, 44:8‐PI, 42:7‐, 44:5‐PA, 44:5‐PS, 34:2‐PE, and 34:0‐DAG), three sphingolipids (38:2‐GlcCer, 36:1‐GlcA‐β‐Cer, and N‐tetradecanoyl‐sphing‐4‐enine‐1‐2‐aminoethylphosphonate), and five unknown metabolites (Figure 1d). The JA‐dependent accumulation of β‐d‐glucan in response to BPH attracted our attention because glucan is widely involved in plant‐insect interactions (Calderon‐Cortes *et al*., 2012).

Mixed‐linkage β‐1,3;1,4‐d‐glucans (MLGs) are Poaceae‐specific β‐d‐glucans that are essential for several developmental and defence processes in rice (Burton and Fincher, 2009; Zhang *et al*., 2021). Therefore, we measured MLG contents in NIP, *oslox2*, and *oscoi1* upon BPH feeding for 0 and 24 h, using endo‐hydrolase‐based high performance anion exchange chromatography (HPAEC) and quantitative assay kits. MLG levels increased in NIP after 24 h of BPH infestation (Figure 1e,f). Consistent with this finding, the BPH‐responsive accumulation of MLG was significantly reduced in both *oslox2* and *oscoi1* compared to NIP (Figure 1e,f). To further validate the functional link between MLGs and the JA pathway, we performed a chemical complementation test of *oslox2* and *oscoi1* plants via exogenous application of 50 ppm MLGs. As expected, the BPH‐sensitive phenotypes of the *oslox2* and *oscoi1* mutants were recovered by MLG treatment compared to the untreated controls (Figure S3e,f).

### Overproduction of MLGs enhances BPH resistance in rice

MLGs are primarily synthesized by members of the cellulose synthase‐like CslF family (Burton *et al*., 2006; Lim *et al*., 2018). Genetic analyses suggested that the loss of *OsCslF6* leads to significant decreases in MLG levels in rice cell walls (Vega‐Sanchez *et al*., 2012). To identify the *OsCslF* genes involved in BPH‐induced MLG accumulation, we examined the spatial and temporal expression patterns of various *OsCslF* gene family members. *OsCslF2* and *OsCslF6* were mainly expressed in leaf sheaths and stems, while *OsCslF1*, *OsCslF3*, *OsCslF4*, *OsCslF7*, *OsCslF8*, and *OsCslF9* were abundantly expressed in roots (Figure S5a). Upon BPH exposure, most *OsCslF* genes (except for *OsCslF9*) were upregulated at 6 h after BPH infestation, with peak expression detected at 12 or 24 h after treatment (Figure S5b). Among these genes, *OsCslF6* transcript levels were significantly lower in the *oslox2* and *oscoi1* mutants compared to NIP (Figure S5b). Moreover, at 6, 12 and 24 h after treatment with 100 μm MeJA, the transcript levels of *OsCslF6* were induced, but at significantly lower levels in the *oscoi1* mutants compared to wild‐type NIP and *oslox2* plants (Figure S5c). These findings suggest that *OsCslF6* plays a major role in MLG biosynthesis in the leaf sheaths and stems, which are the sites of BPH infestation in rice.

**Figure 2:**
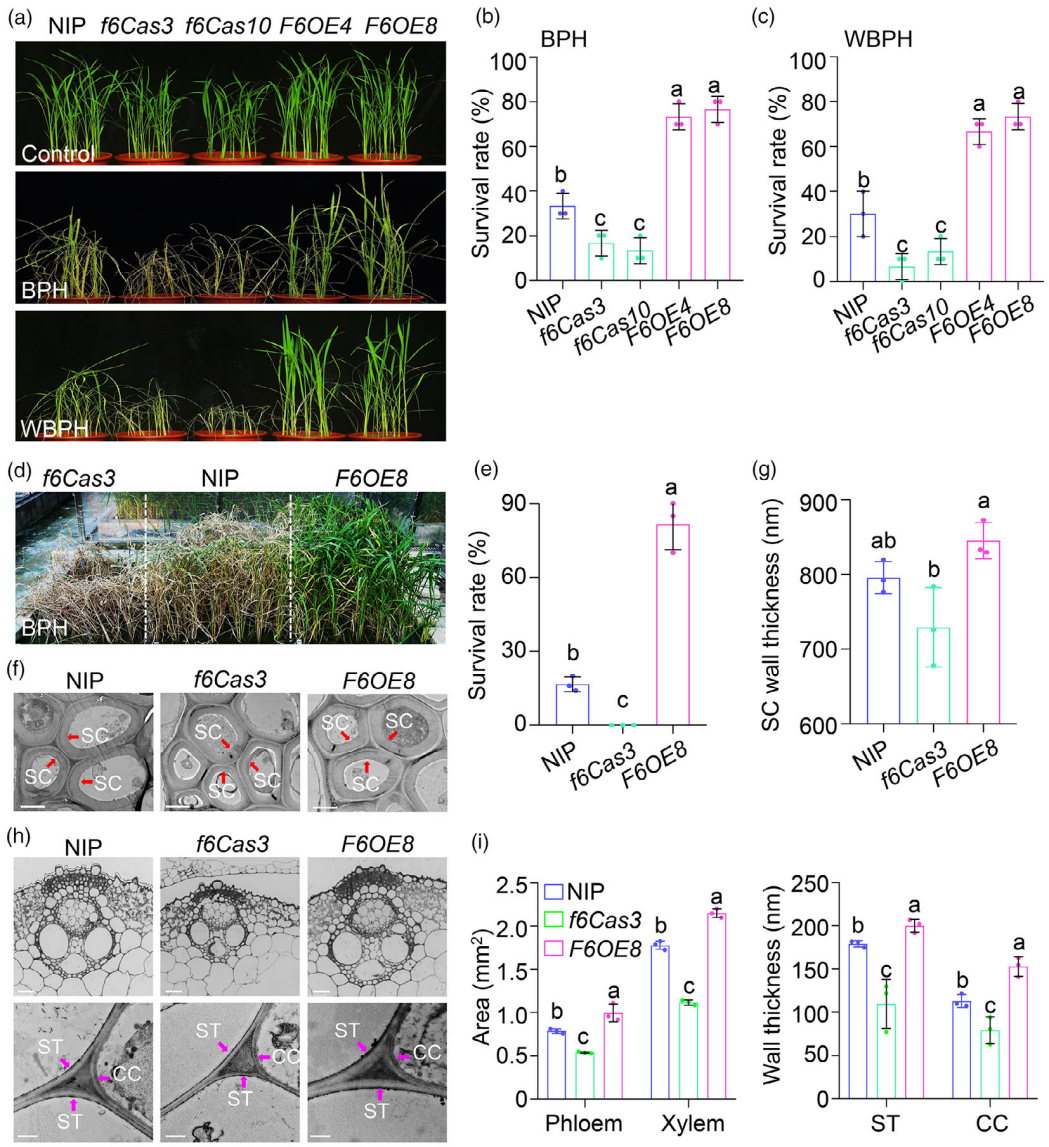
MLGs enhance BPH resistance and cell wall thickness in rice. (a) Phenotypes of NIP, *oscslf6* mutants (*f6Cas3* and *f6Cas10*), and *OsCslF6*‐overexpressing (*F6OE4* and *F6OE8*) plants infested with BPH and WBPH for 7 and 8 days, respectively. (b, c) Survival rates of various genotypes infested with BPH (b) or WBPH (c) in (a). The survival rates were determined after treatment at BPH or WBPH for 7 and 8 days, respectively. Data are means ± SD (*n* = 3 biological replicates). For each replicate, 10 plants per genotype were used for the calculation. (d) Phenotypes of 6‐week‐old NIP, *f6Cas3*, and *F6OE8* plants infested with BPH for 15 days in the field. (e) Survival rates of various genotypes infested with BPH shown in (d). Data are means ± SD (*n* = 3 biological replicates). For each replicate, 40 plants per genotype were used for the calculation. (f) Transmission electron micrograph showing sclerenchyma cells in leaf sheaths and stems of NIP, *f6Cas3*, and *F6OE8* plants. SC, sclerenchyma cells. Scale bars, 500 nm. (g) Quantification of cell wall of sclerenchyma cells in leaf sheaths and stems of NIP, *f6Cas3*, and *F6OE8* plants. Data are means ± SD (*n* = 3 biological replicates; for each replicate, 15 cells from five individual sections per genotype were used for quantification). (h) Confocal micrographs and transmission electron micrographs (TEM) showing vascular bundles, sieve tubes and companion cells in leaf sheaths and stems of NIP, *f6Cas3*, and *F6OE8* plants. Scale bars in confocal micrographs, 25 mm; Scale bars in TEM micrographs, 500 nm. (i) Quantification of phloem and xylem area and cell wall thickness of sieve tubes and companion cells in leaf sheaths and stems of NIP, *f6Cas3*, and *F6OE8* plants. Data are means ± SD (*n* = 3 biological replicates; for each replicate, 15 cells from five individual sections per genotype were used for quantification). Letters indicate significant differences between groups conducted by one‐way ANOVA, *P* < 0.05.

To verify the role of *OsCslF6* in BPH resistance, we generated *OsCslF6* knockout (*f6Cas3* and *f6Cas10*) (Figure S6a) and *OsCslF6* overexpression (*F6OE4* and *F6OE8*) transgenic lines (Figure S6b). Four‐week‐old *f6Cas3* and *f6Cas10* plants showed dwarf phenotypes compared to wild‐type plants, which is consistent with previous findings (Vega‐Sanchez *et al*., 2012). *F6OE4* and *F6OE8* plants displayed stronger morphological changes than NIP at this stage (Figure 2a). Insect feeding assays indicated that under growth room conditions, the *f6Cas3* and *f6Cas10* mutants were hypersensitive to 7 days of BPH infestation compared to wild‐type NIP plants (Figure 2a), based on survival rates (Figure 2b). By contrast, compared to NIP and *f6Cas* plants, the *F6OE4* and *F6OE8* lines were more resistant to BPH (Figure 2a,b). After 7 days of BPH feeding, only 40% of wild‐type plants survived, whereas more than 75% of *OsCslF6*‐overexpressing plants survived (Figure 2b). To determine the potential role of OsCslF6 in broad‐spectrum resistance to planthoppers, we tested the phenotype of *f6cas9* and *F6OE* plants with another phloem‐sucking herbivore, white‐backed planthopper (WBPH, *Sogatella furcifera* Horvath). Compared with NIP, the *f6cas9* mutants were more susceptible to WBPH, while the *F6OE* lines were more tolerant to WBPH feeding (Figure 2a,c).

To further confirm the role of *OsCslF6* in JA‐mediated defence against insects, we exposed 6‐week‐old field‐grown NIP, *f6Cas3*, and *F6OE8* plants to BPH. Consistent with the data from plants in the growth room (Figure 2a,b), compared to wild‐type plants, the *f6Cas3* mutant was more sensitive and the *F6OE8* plants were more resistant to BPH feeding in the field (Figure 2d), based on survival rates (Figure 2e). Analysis using a quantitative assay kit revealed that MLG levels were much lower in the *f6Cas3* and *f6Cas10* mutants but significantly higher in the *F6OE4* and *F6OE8* lines compared to NIP (Figure S6c). These findings suggest that *OsCslF6* is important for resistance to BPH infestation.

To investigate the structural mechanism by which OsCslF6 affects insect resistance, we used transmission electron microscopy and confocal microscopy to compare the vascular bundle size and cell wall thickness in transverse sections of leaf sheaths from rice plants of different genotypes at the tillering stage. We observed that the wall thickness of sclerenchyma cells of *f6Cas3* and *F6OE8* plants did not significantly differ from that of NIP plants (Figure 2f,g). However, the vascular bundles of *f6Cas3* were significantly smaller than those of wild‐type plants, whereas the vascular bundles of *F6OE8* plants were significantly larger (Figure 2h), as confirmed by measuring the areas of phloem and xylem (Figure 2i). Transmission electron microscopy showed that the cell walls of sieve tubes and companion cells were significantly thinner in the *f6Cas3* mutants, but significantly thicker in *F6OE8* plants, compared to NIP (Figure 2h,i). These findings suggest that overexpressing *OsCslF6* enhances cell wall thickness in vascular bundles, which contributes to improved resistance to insect herbivory.

### OsMYC2 regulates *OsCslF6* transcript levels and BPH resistance

AtMYC2 is a key transcription factor that is essential for JA signalling in Arabidopsis (Lorenzo *et al*., 2004). To better understand the molecular link between JAs and OsCslF6‐mediated BPH resistance in rice, we generated *OsMYC2* knockdown (*OsMYC2 RNAi‐1* and *OsMYC2 RNAi‐2*) and overexpression (*OsMYC2 OE‐1* and *OsMYC2 OE‐2*) transgenic lines (Figure S7a,b). We then measured *OsCslF6* transcript levels in *OsMYC2 RNAi* and *OsMYC2‐OE* plants by RT‐qPCR. Before BPH exposure, *OsCslF6* transcript levels were significantly higher in *OsMYC2‐OE* seedlings than in wild‐type NIP and after BPH exposure, *OsCslF6* transcript levels were lower or higher in *OsMYC2 RNAi* and *OsMYC2‐OE* seedlings than in NIP, respectively (Figure S7c). Consistent with their *OsCslF6* transcript levels, *OsMYC2‐OE* and *OsMYC2 RNAi* seedlings had significantly higher or lower MLG contents than the wild type upon BPH treatment, respectively (Figure S7d).

**Figure 3:**
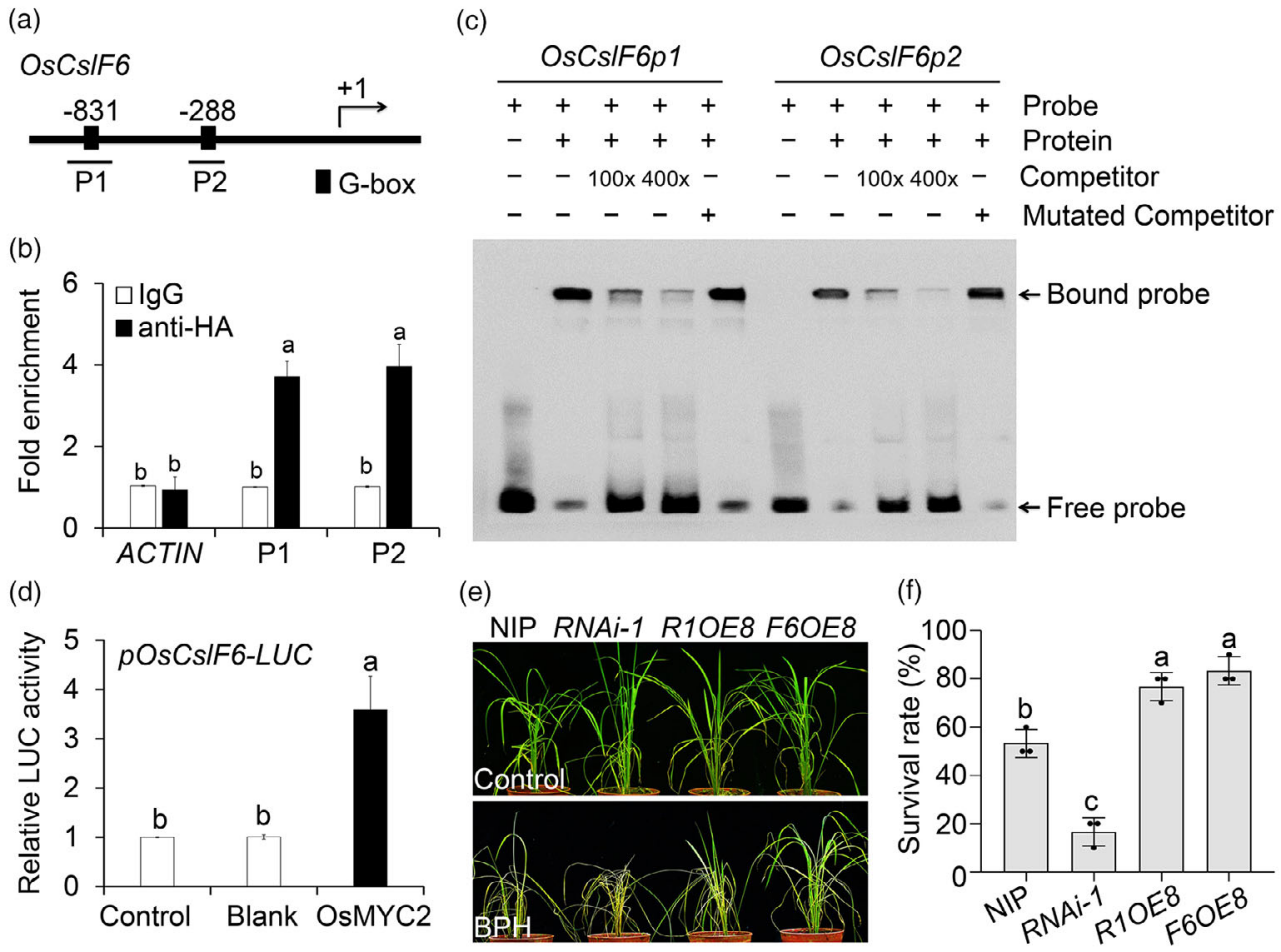
The OsMYC2‐OsCslF6 module contributes to BPH resistance. (a) Schematic diagram of the potential G‐box motifs in the *OsCslF6* promoter. Numbers indicate the nucleotide positions relative to their corresponding translational start site, which is shown as +1. (b) ChIP‐qPCR data showing the promoter fragments P1 (−878 to −767) and P2 (−314 to −215) that were amplified from immunoprecipitated proteins pulled down by anti‐HA antibodies. The *OsACTIN1* promoter was used as a control. Data are means ± SD (*n* = 3 biological replicates). (c) EMSA showing the binding of OsMYC2 to the G‐box elements in P1 and P2 in the *OsCslF6* promoter. Free and bound DNAs (arrows) were separated in an acrylamide gel. Excess cold, unlabeled probes were used as competitors (lanes 3 and 4), and mutated probes (lane 5) were produced by replacing the G‐box motifs. (d) Luciferase activity in rice protoplasts co‐transfected with the effector plasmids (OsMYC2 and vector control) and their combinations with reporter *pOsCslF6‐LUC*. The activity of protoplasts transfected with the empty effector construct (control) was defined as 1. Data are means ± SD (*n* = 3 biological replicates). (e) Phenotypes of NIP, *OsMYC2‐RNAi* (*RNAi‐1*), *OsMYC2‐RNAi F6OE8* (*R1OE8*; generated by crossing an *OsMYC2‐RNAi* plant to the *F6OE8* line), and *F6OE8* infested with BPH for 6 days. (f) Survival rates of NIP, *RNAi‐1*, *R1OE8*, and *F6OE‐8* plants infested with BPH in (e). The survival rate was determined after treatment with BPH for 6 days. Data are means ± SD (*n* = 3 biological replicates; 10 plants were used for each replicate). Letters indicate significant differences between groups conducted by one‐way ANOVA, *P* < 0.05.

In Arabidopsis, AtMYC2 binds to G‐box or G‐box‐like motifs in the promoters of JA‐responsive genes to directly regulate their transcription (Dombrecht *et al*., 2007; Kazan and Manners, 2013). Bioinformatic analysis of the promoters of the *OsCslF* genes revealed two putative G‐box elements in the promoter region of *OsCslF6* (Figure 3a) and one or two G‐box or G‐box like elements in the promoters of other *OsCslF* genes (Figure S8a). In chromatin immunoprecipitation‐quantitative PCR (ChIP‐qPCR) assays, OsMYC2 bound to both G‐box elements in the *OsCslF6* promoter (Figure 3b), but not the other *OsCslF* promoters (Figure S8b). Electrophoretic mobility shift assays (EMSAs) confirmed that the G‐boxes located at P1 and P2 of the *OsCslF6* promoters are targets of OsMYC2 (Figure 3c). The addition of unlabeled competitor probes markedly reduced the signal intensities of OsMYC2–P1/P2 complexes; this effect was diminished in reactions using mutant competitor probes (Figure 3c). Finally, in dual‐luciferase assays, co‐expression with OsMYC2 significantly increased the activity of luciferase driven by the *OsCslF6* promoter compared to the controls (Figure 3d and Figure S8c). These results suggest that OsMYC2 binds directly to the *OsCslF6* promoter to control BPH‐induced MLG accumulation.

To genetically link OsMYC2 to the cellular function of *OsCslF6*, we crossed *OsMYC2 RNAi‐1* with *F6OE8* plants to generate *OsMYC2‐RNAi OsCslF6‐OE* double transgenic plants (designated *R1OE8*). As shown in Figure 3, overexpressing *OsCslF6* rescued the increased BPH sensitivity of *OsMYC2 RNAi‐1* plants (Figure 3e,f), suggesting that OsMYC2 plays an indispensable role in regulating *OsCslF6*‐mediated plant responses to BPH infestation. Consistent with their increased MLG contents, the two *OsMYC2‐OE* lines exhibited enhanced tolerance to BPH feeding compared to the NIP control (Figures S9a,b).

### Natural variation in the *OsCslF6* promoter affects MLG‐mediated BPH resistance

**Figure 4:**
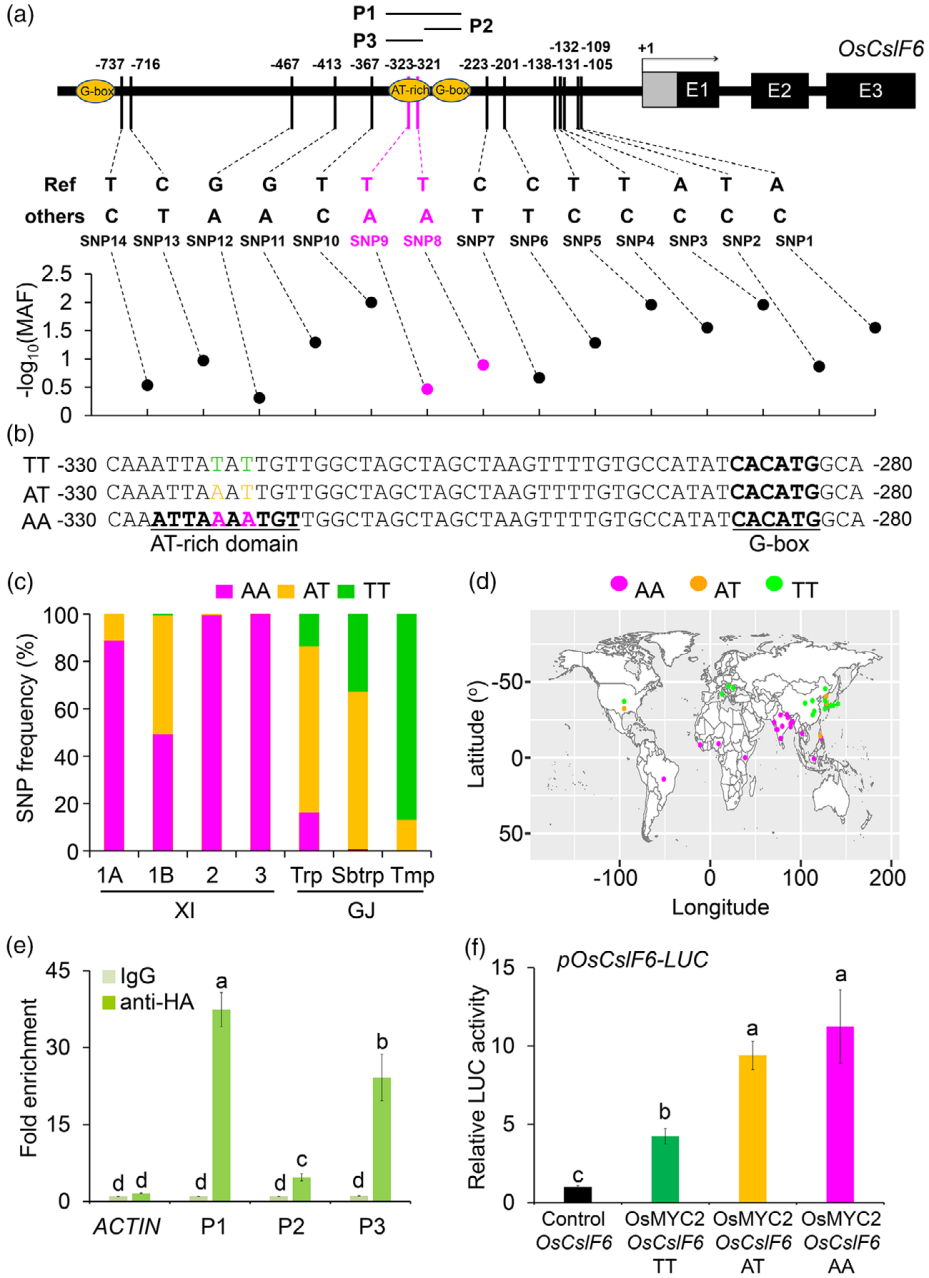
SNP8 and SNP9 in the *OsCslF6* promoter are associated with its transcription activity. (a) SNP analysis of the *OsCslF6* promoter in 3K‐sequenced accessions. SNP8 and SNP9 variations are indicated by bold magenta font. Minor Allele Frequency (MAF > 0.01) analysis of 14 variants in the *OsCslF6* promoter region is shown below the diagram. (b) Sequence alignment showing nucleotide variations in the AT‐rich box of the *OsCslF6* promoter. The TT, AT, and AA alleles are indicated by bold green, yellow, and magenta font, respectively. (c) Distribution of SNP8 and SNP9 in the *indica* (XI‐1A, 1B, 2, and 3) and *japonica* (GJ‐Trp, GJ‐Sbtrp, and GJ‐Tmp) subpopulations. (d) Worldwide locations of SNP8 and SNP9 in the *OsCslF6* promoter. The size of the pie chart is proportional to the number of rice varieties in 3K‐sequenced accessions. Varieties with the AA allele are primarily grown in tropical regions, whereas varieties with the TT allele are primarily grown in temperate regions. (e) ChIP‐qPCR data showing the promoter fragments P1 (−415 to −215), P2 (−314 to  −215), and P3 (−415 to −306) in (a; TT version) that were amplified from immunoprecipitated proteins pulled down by anti‐HA antibodies. The *OsACTIN1* promoter was used as a control. Data are means ± SD (*n* = 3 biological replicates). (f) Luciferase activity of rice protoplasts co‐transfected with the effector plasmids (OsMYC2 and vector control) combined with reporters (control+*OsCslF6*; OsMYC2 + *OsCslF6* TT; OsMYC2 + *OsCslF6* AT; and OsMYC2 + *OsCslF6* AA). The activity of protoplasts transfected with the empty effector construct was defined as 1. Data are means ± SD (*n* = 3 biological replicates). Letters indicate significant differences between groups conducted by one‐way ANOVA, *P* < 0.05.

To investigate the possible contribution of natural variation in *OsCslF6* to MLG‐mediated BPH resistance in rice, we analysed the single‐nucleotide polymorphisms (SNPs) in the promoter and coding regions of *OsCslF6* from the 3K Rice Genomes Project (Wang *et al*., 2020). We did not detect nonsynonymous sequence variants in the *OsCslF6* coding region; however, we identified 14 SNPs in its promoter (Figure 4a).

An analysis of regulatory elements suggested the potential importance of SNP8 (T/A alleles) and SNP9 (T/A alleles) at positions −321 and −323, respectively, in the *OsCslF6* promoter (Figure 4a,b). Bioinformatic analyses suggested that the sequence variants with the AA alleles for SNPs 8 and 9, but not the AT and TT alleles, contained an AT‐rich domain (Figure 4b). This DNA‐binding domain is conserved in all eukaryotes and is targeted by AT‐Rich Interaction Domain (ARID)‐containing transcription factors (Xu *et al*., 2015; Zheng *et al*., 2014). Indeed, RT‐qPCR analyses showed that all six rice *ARID* genes, *OsARID1* to *OsARID6*, were transcriptionally upregulated by BPH feeding (Figure S10), supporting their potential involvement in regulating insect defence responses in plants.

We further analysed the distribution of the TT, AT, and AA alleles of SNP8 and SNP9 in the cultivated rice species from the 3K Rice Genomes Project. The AA allele was mainly found in *indica* rice, the TT allele was mainly found in *japonica* rice (Figure 4c), and the AT allele was present in both cultivars (Figure 4c). Geographical localization analysis revealed that varieties with the AA allele were primarily distributed in tropical regions, whereas varieties with the TT allele were primarily distributed in temperate regions (Figure 4d).

Given that the AT‐rich motif in the AA allele promoter is very close to the OsMYC2‐binding G‐box element (Figure 4b), we next explored the potential link between these two elements in regulating the transcription of *OsCslF6*. ChIP‐qPCR assays revealed that compared to the G‐box element alone, the presence of the AT‐rich element strongly activated the association of OsMYC2 with the G‐box element in the *OsCslF6* promoter (Figure 4e). Furthermore, luciferase activity assays showed that either the single mutation of SNP8 (TT to AT) or double mutation of SNP8/9 (TT to AA) significantly enhanced the OsMYC2‐induced activation of *OsCslF6* expression compared to the allele (Figure 4f). These results suggest that SNP8 and SNP9 in the *OsCslF6* promoter likely affect its regulation.

**Figure 5:**
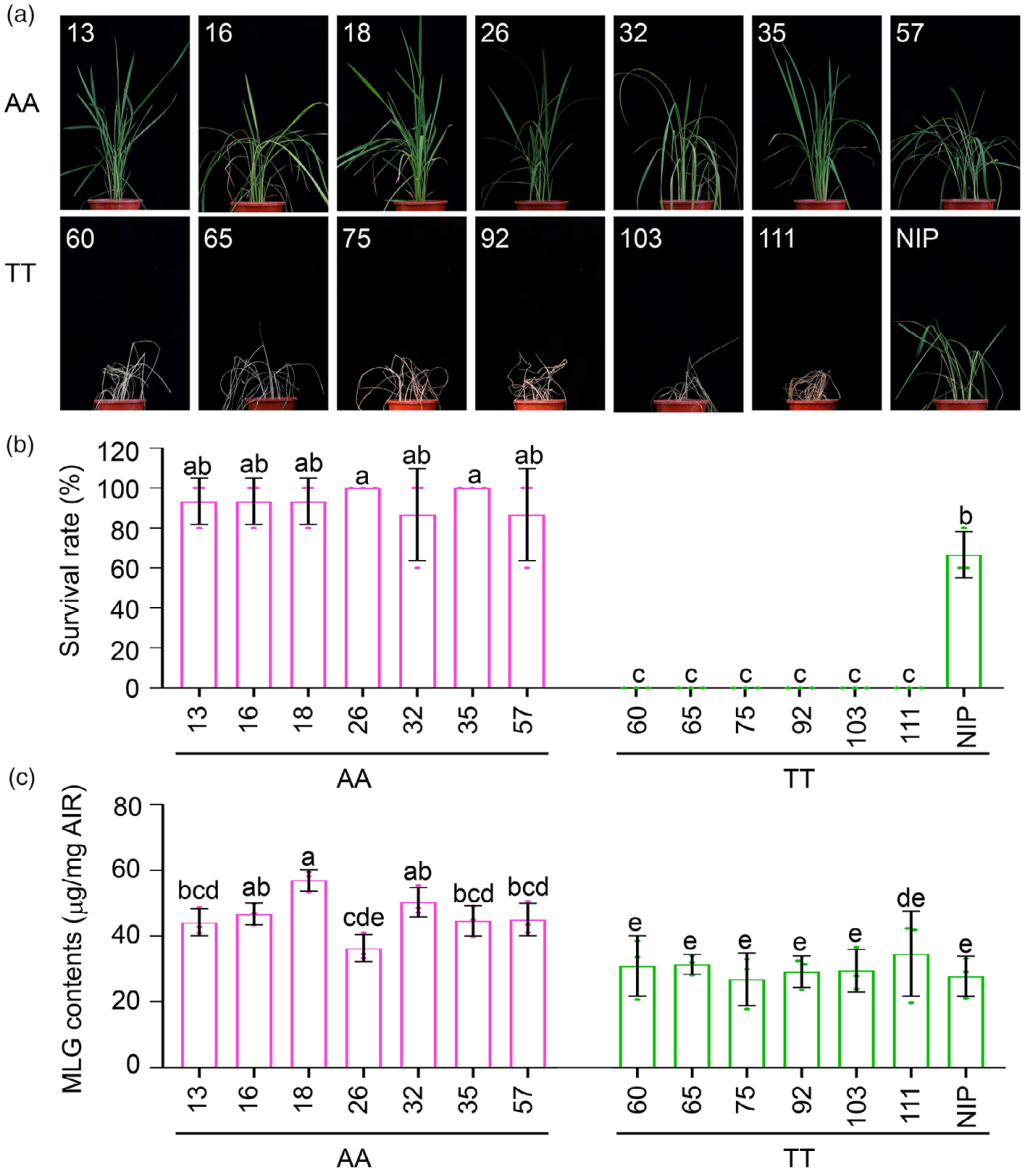
Natural variation of *OsCslF6* confers MLG‐mediated BPH resistance. (a, b) Phenotypes (a) and survival rates (b) of rice accessions with the AA allele (13, 16, 18, 26, 32, 35, and 57) and TT allele (60, 65, 75, 92, 103, 111, and NIP) infested with BPH for 13 days. The survival rate was determined after 13 days of BPH treatment. Data are means ± SD (*n* = 3 biological replicates; 10 plants were used for each replicate). (c) MLG contents of rice subpopulations with the AA or TT allele under normal conditions. Data are means ± SD (*n* = 3 biological replicates). Letters indicate significant differences between groups conducted by one‐way ANOVA, *P* < 0.05.

To further test the effect of these SNPs on *OsCslF6* function in BPH resistance, we randomly selected seven cultivars with the AA or TT allele from the 3K sequenced rice accessions for BPH feeding tests. Most accessions containing the AA allele showed more resistance after 13 days of BPH infestation than accessions containing the TT allele under natural growth conditions (Figure 5a), based on the survival rate of each accession (Figure 5b). Consistent with these BPH resistance phenotypes, most of the AA accessions also accumulated more MLGs than the TT accessions under normal conditions (Figure 5c). These findings demonstrate that SNP8 and SNP9 in the *OsCslF6* promoter contribute to MLG‐mediated BPH resistance in rice.

### MLGs bind OsLecRK1 *in vitro* and stimulate its activity *in vivo*

**Figure 6:**
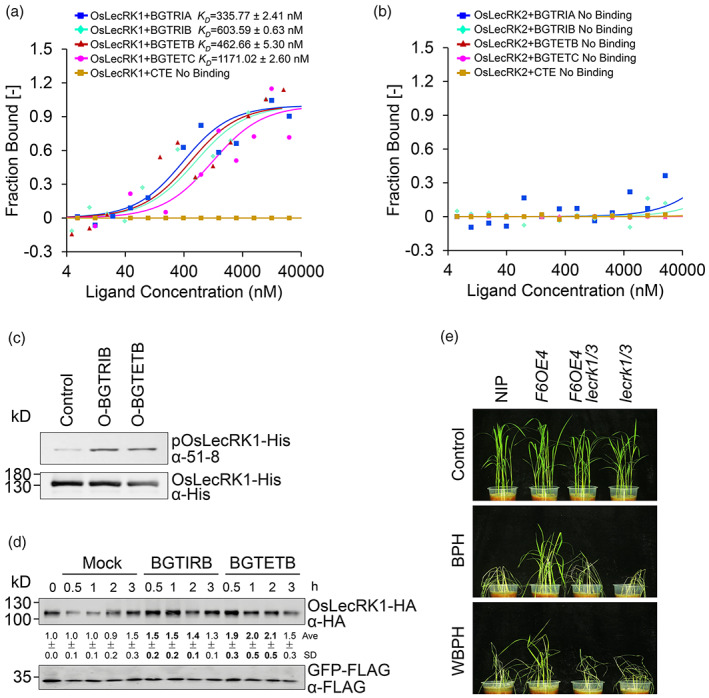
MLGs bind OsLecRK1 *in vitro* and stimulate its activity to enhance BPH resistance. (a, b) Binding analyses of OsLecRK1‐His (a) and OsLecRK2‐His (b) recombinant proteins with the oligosaccharides using microscale thermophoresis (MST) assays. BGTRIA, BGTRIB, BGTETB, and BGTETC were used as ligands titrated with OsLecRK1 and OsLecRK2 proteins. CTE was used as a negative control. *K*_D_, dissociation constant. BGTRIA, 3^2^‐β‐d‐glucosyl‐cellobiose, G3G4G. BGTRIB, 3^1^‐β‐d‐cellobiosyl‐glucose, G4G3G. BGTETB, 3^1^‐β‐d‐cellotriosyl‐glucose, G4G4G3G. BGTETC, 3^2^‐β‐d‐Cellotriosyl‐cellobiose +3^3^‐β‐d‐glucosyl‐cellotriose, G4G3G4G + G3G4G4G. CTE, cellotetraose, G4G4G4G. (c, d) *in vitro* (c) and *in vivo* (d) kinase assays showing the stimulation of OsLecRK1 activity by BGTRIB and BGTETB. (c) *in vitro* OsLecRK1‐His kinase activity was detected with thiophosphate ester‐specific 51‐8 antibody. OsLecRK1‐His recombinant proteins were detected with anti‐His antibody as a loading control. (d) The protoplast cells isolated from wild‐type NIP plants were treated with sterile water (Mock), 10 μm BGTRIB, and 10 μm BGTETB, then harvested at 0, 0.5, 1, 2, and 3 h. The relative intensities of OsLecRK1‐HA proteins were measured by normalizing to the intensities of corresponding co‐transfected GFP‐FLAG and compared to that of 0 h control. Data are means ± SD (*n* = 3 biological replicates). (e) Phenotypes of 3‐week‐old NIP, *F6OE4*, *F6OE4 lecrk1/3*, and *lecrk1/3* plants infested with BPH (middle image) and WBPH (bottom image) for 6 and 8 days, respectively.

MLGs are carbohydrate‐based molecular patterns that can be perceived by plants to trigger pathogen‐induced immune responses (Rebaque *et al*., 2021). Moreover, in response to *Magnaporthe oryzae* infection of rice, oligosaccharides released from MLGs act as DAMPs, which are perceived by OsCERK1 to activate immune signalling (Yang *et al*., 2021). We therefore hypothesized that MLG may also function as precursors of DAMPs that are recognized by cell surface OsLecRKs during rice‐BPH interactions. To test this possibility, we first evaluated the association between MLG‐derived oligosaccharides and OsLecRKs by using the recombinant MBP‐OsLecRK1‐His and MBP‐OsLecRK2‐His proteins, as well as various MLG‐based compounds, specifically 3^2^‐β‐d‐glucosyl‐cellobiose (BGTRIA, G3G4G), 3^1^‐β‐d‐cellobiosyl‐glucose (BGTRIB, G4G3G), 3^1^‐β‐d‐cellotriosyl‐glucose (BGTETB, G4G4G3G), and BGTETC (3^2^‐β‐d‐Cellotriosyl‐cellobiose + 3^3^‐β‐d‐glucosyl‐cellotriose, G4G3G4G + G3G4G4G). Microscale thermophoresis (MST) assays showed that the MBP‐OsLecRK1‐His recombinant protein bound BGTRIA, BGTRIB, BGTETB, and BGTETC with high affinities, as reflected by their disassociation constants (*K*_D_) of 0.34, 0.60, 0.46, and 1.17 μm, respectively (Figure 6a). As a control, the MBP‐OsLecRK1‐His recombinant protein did not bind to the β‐1,4‐glucose‐based cellotetraose (CTE, G4G4G4G; Figure 6a). When we tested the binding of MBP‐OsLecRK2‐His recombinant protein with various oligosaccharides, neither ligand was bound by MBP‐OsLecRK2‐His recombinant protein (Figure 6b).

To confirm that MLGs participate in OsLecRK1‐mediated defence signalling, we investigated the phosphorylation of the OsLecRK1 kinase in the presence or absence of MLG‐based oligosaccharides *in vitro*. As shown in Figure 6c, compared to the mock control, BGTRIB and BGTETB stimulated phosphorylation of MBP‐OsLecRK1‐His recombinant protein, as detected by the anti‐51‐8 antibody. To examine whether MLGs activate OsLecRK1 *in vivo*, we treated protoplasts isolated from NIP with sterile water, 10 μm BGTRIB, and BGTETB, and harvested the protoplasts at 0, 0.5, 1, 2, and 3 h after treatment. Immunoblot analyses showed that the abundance of OsLecRK1‐HA fusion protein increased upon BGTRIB and BGTETB exposure from 0.5 to 3 h and peaked at 0.5 or 1 h (Figure 6d), suggesting that MLGs rapidly activate the accumulation of OsLecRK1 *in vivo*.

To further examine the genetic relationship between OsCslF6 and OsLecRK1 during the rice‐BPH interaction, we generated the *F6OE4 oslecrk1/3* line by CRISPR‐mediated knockout of *OsLecRK1* and *OsLecRK3* in the *F6OE4* background. Phenotypic analyses showed that, in contrast to the improved growth of *F6OE4* line, the 3‐week‐old seedlings of the *F6OE4 oslecrk1/3* and *oslecrk1/3* lines were smaller in size than the wild‐type NIP plants under normal growth conditions (Figure 6e). Upon BPH feeding for 6 days, the *oslecrk1/3* mutant was hypersensitive to BPH attack (Figure 6e), consisting with previous findings (Liu *et al*., 2015). In contrast, the *F6OE4 oslecrk1/3* plants were also susceptible to BPH feeding, which abolished the enhanced tolerance seen in the *F6OE4* line (Figure 6e). In addition, the enhanced resistance to WBPH of *F6OE4* lines was abolished in *F6OE4 oslecrk1/3*, which was as susceptible to WBPH feeding as the *oslecrk1/3* mutant (Figure 6e). These findings suggest that OsCslF6‐mediated MLG biosynthesis acts upstream of OsLecRK1 in the rice response to pest infestation. Taken together, our results reveal that MLG‐derived oligosaccharides bind to and stimulate OsLecRK1 during rice‐BPH interaction.

## Discussion

The cell wall is primarily composed of polysaccharides including cellulose (β‐1,4‐d‐glucose), callose (β‐1,3‐d‐glucose), and MLGs. This structure serves as the first barrier defending plants against pest and pathogen invasion (Calderon‐Cortes *et al*., 2012; Vorwerk *et al*., 2004). BPH is an herbivore that feeds on phloem sap in leaf sheaths through its stylet; therefore, the thickness and mechanical strength of cell walls are crucial for plant resistance to BPH (Guo *et al*., 2018). In response to planthoppers, callose synthase genes are upregulated in rice to enhance callose deposition in sieve tubes, preventing the insect from inserting its stylet and ingesting sap (Calderon‐Cortes *et al*., 2012). If the insect manages to feed, the resulting cell wall fragments may act as DAMPs to stimulate cell surface‐localized receptor proteins, thus initiating defence signalling (Erb and Reymond, 2019).

In this study, we discovered that the MLGs produced by OsCslF6 integrate both mechanisms by reinforcing cell wall thickness in vascular bundles and stimulating OsLecRK1‐mediated defence signalling during the rice‐BPH interaction. MLGs are a class of Poaceae‐specific metabolites (Burton and Fincher, 2009; Ermawar *et al*., 2015), and it is conceivable that (like GSs in Arabidopsis) MLGs function downstream of JAs to help regulate defence responses against pests in plants of the Poaceae family, including rice.

In Arabidopsis only one AtCOI1 co‐receptor is involved in perceiving JA (Wang *et al*., 2019); by contrast, rice has three: OsCOI1a, OsCOI1b, and OsCOI2 (Lee *et al*., 2015). Among these proteins, OsCOI1a and OsCOI1b have higher sequence identity (>80%) and are proposed have central functions in JA signalling (Lee *et al*., 2015). Consistent with this notion, our results showed that two *oscoi1‐1a/b* double mutants displayed increased sensitivity to BPH feeding (Figure S3) and decreased levels of MLGs (Figure 1f). We also observed that *oscoi1‐1a/b* double mutants retained some sensitivity to MeJA (Figure S2d,e); this may be due to the mutations we used being weak alleles harbouring early stop codons that lead to truncated proteins at positions 451 and 457 in OsCOI1a, and 424 and 419 in OsCOI1b, respectively (Figure S2). Alternatively, COI2 may share a redundant function in the *oscoi1‐1a/b* double mutants.

Enhancing of physical barriers, including the sclerenchyma and vasculature, can prevent BPH stylets from reaching the phloem cells for feeding. In rice leaf sheaths, the sclerenchyma is located under the epidermis and above the vascular bundles. *Bph3*, *Bph6*, *Bph9*, *Bph14*, and *Bph26*, are strongly expressed in the vascular bundle cells (Du *et al*., 2009; Guo *et al*., 2018; Liu *et al*., 2015; Tamura *et al*., 2014; Zhao *et al*., 2016), although we do not know whether these genes regulate phloem strength in response to BPH feeding in rice. *Bph30* is a rice BPH resistance gene encoding a novel protein with two leucine‐rich domain that fortifies sclerenchyma cells to prevent BPH stylets from reaching the phloem (Shi *et al*., 2021), supporting the importance of sclerenchyma‐mediated structural mechanisms in BPH resistance. Our results showed that OsCslF6‐catalysed MLG deposition enhances BPH resistance by specifically modulating the cell wall thickness of vascular bundles, but not the sclerenchyma cells (Figure 2). Like *Bph30*, the *OsCslF6*‐overexpressing lines are also resistant to WBPH (Figure 2a), indicating that fortifying the sclerenchyma or vascular bundle cells produces broad‐spectrum pest resistance in rice.

Insect feeding or pathogen infection disrupts cell wall integrity; this mechanical damage is perceived by the plasma membrane‐localized pattern‐recognition receptors (PRRs; Reymond, 2021). In microbial infection, cell wall fragments are released into the extracellular space, where they act as DAMPs to bind to PRRs, eventually leading to immune responses and pathogen resistance (Gong *et al*., 2020; Gust *et al*., 2017; Reymond, 2021). OsLecRKs are cell surface PRRs conferring strong and broad‐spectrum resistance to planthoppers in rice (Cheng *et al*., 2013a; Liu *et al*., 2015). Nevertheless, the biochemical mechanism by which OsLecRKs bind to cell wall components and contribute to DAMP‐mediated immunity responses is unknown.

Using MST and kinase activity assays, we established that the MLG‐based oligosaccharides bind to recombinant OsLecRK1 protein with high affinities *in vitro* and activate its kinase activity *in vivo* (Figure 6a–d). Moreover, genetic analysis showed that deletion of *OsLecRK1* and *OsLecRK3* abolished the MLG‐mediated BPH and WBPH resistance phenotypes of the *OsCslF6*‐overexpressors (Figure 6e), suggesting that OsLecRK1 signalling is indeed downstream of MLG biosynthesis during the rice‐planthopper interaction. Thus, our results imply that MLG‐derived oligosaccharides may act as DAMPs and are likely recognized by OsLecRK1, which induces downstream defence responses. However, it is still unclear whether the MLG‐based oligosaccharides are cleaved by a specific rice endoglucanase, or an endoglucanase secreted in pest saliva. A recent study showed that during infection of rice, the fungal pathogen *Magnaporthe oryzae* secretes two endoglucanases, MoCel12A and MoCel12B, which release MLG‐based oligosaccharides that are perceived by the OsCERK1 immune complex (Yang *et al*., 2021). To the best of our knowledge, no previous studies have identified salivary endo‐β‐1,3;1,4‐glucanase in planthoppers, including BPH. Alternatively, MLG may function in rice‐BPH interactions in an OsLecRK1‐independent manner; elucidating the signalling cascades downstream of OsLecRK1 will clarify whether this occurs. Thus, further investigation of the specific enzymes in distinct BPH subspecies and the functional kinase substrates of OsLecRK1 would increase our understanding of the molecular mechanisms underlying rice‐BPH interactions.

Previous genome‐wide association analyses identified 3502 SNPs and 59 loci associated with BPH resistance in rice (Zhou *et al*., 2021), which may have important implications for the control of BPH. Here, we observed that SNP8 and SNP9 in the *OsCslF6* promoter produced an AT‐rich domain in the AA allele, but not in the AT or TT alleles (Figure 4a,b). This variation is likely associated with MLG deposition and BPH resistance in different cultivars selected from the 3K sequenced rice accessions (Figure 5). It is worth noting that BPH cannot survive in temperate regions; the northern geographic limit of BPH overwintering is approximately 23–25°N (Jing *et al*., 2017; Xue *et al*., 2014; Zheng *et al*., 2021). Despite its capability for long‐distance flight, the line between the overwintering vs. non‐overwintering of BPH likely overlaps with the global distribution of the AA and TT alleles in the *OsCslF6* promoter (Figure 4c,d), suggesting that SNP8/9 were positively selected to enhance MLG deposition and BPH resistance during the co‐evolution of rice and BPH.

In multicellular eukaryotes, the AT‐rich domain is a conserved DNA binding element targeted by ARID transcription factors and has a wide range of functions (Wilsker *et al*., 2005). In plants, the Arabidopsis AtARID1 and rice OsARID3 proteins play important roles in plant developmental processes, including sperm cell formation and shoot meristem development (Xu *et al*., 2015; Zheng *et al*., 2014). Given the importance of this AT‐rich domain in modulating OsMYC2‐activated *OsCslF6* transcription (Figure 4f), we propose that other AT‐rich domain‐binding transcription factors, most likely OsARID proteins, are also involved in regulating MLG deposition in response to BPH attack. Consistent with this hypothesis, we showed that the transcripts of all six rice *OsARID* genes were significantly upregulated upon BPH feeding (Figure S10), suggesting that the OsARID proteins may participate in BPH resistance, although their functional associations with OsCslF6 and MLG biosynthesis remain to be elucidated.

**Figure 7:**
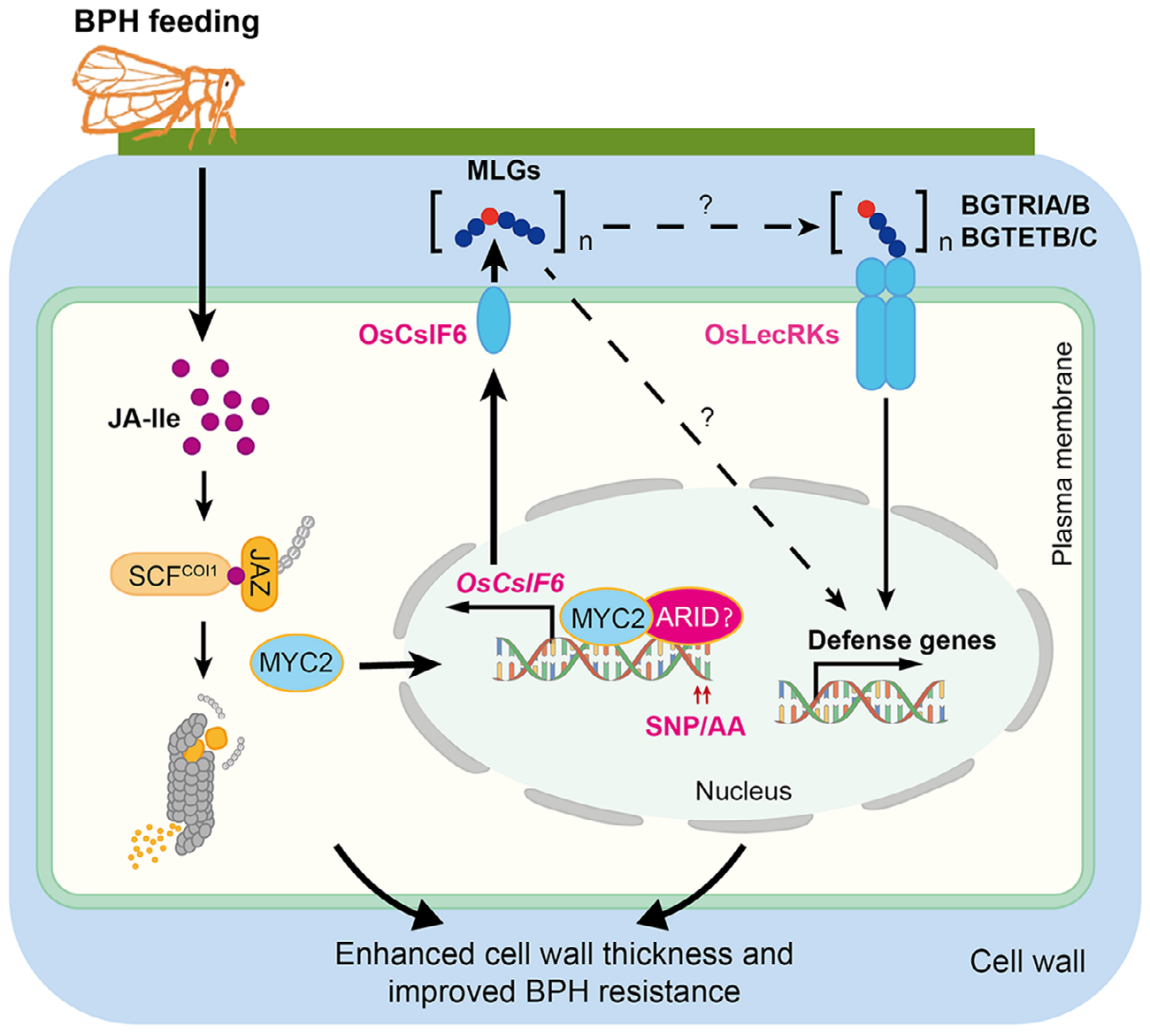
Working model of the role of MLGs in regulating BPH resistance. BPH feeding induces the production of MLG‐derived oligosaccharides such as BGTRIA/B and BGTETB/C, to activate plant defence responses by directly binding to the membrane‐localized OsLecRK receptor kinases, or through an unknown OsLecRK‐independent mechanism. Meanwhile, BPH triggers accumulation of jasmonates (JA‐Ile), which are perceived by the co‐receptors SCF^OsCOI1a/1b^ (SCF^COI1^) and OsJAZ (JAZ). The ubiquitin ligase SCF^COI1^ promotes the ubiquitination of JAZ proteins and guides their proteasomal degradation. The transcription factor OsMYC2 (MYC2), which is inhibited by JAZ proteins under normal growth conditions, is released to activate the expression of *OsCslF6* and the accumulation of MLGs. Natural variants in the AT‐rich motif of the *OsCslF6* promoter (SNP8/9) are positively associated with MLG deposition and BPH resistance.

Finally, we propose a model for the role of OsCslF6‐catalysed MLGs in regulating JA‐mediated resistance in rice (Figure 7). According to our model, BPH feeding induces the accumulation of JAs, which are perceived by the co‐receptors SCF^OsCOI1^ and OsJAZ. The ubiquitin ligase SCF^OsCOI1^ promotes OsJAZ ubiquitination and proteasomal degradation. The transcription factor OsMYC2, which is inhibited by OsJAZ proteins under normal growth conditions, activates the expression of *OsCslF6* and the accumulation of MLGs to enhance cell wall thickness of vascular bundles, improving plant resistance to BPH (Figure 7). At the same time, the MLG‐based oligosaccharides such as BGTRIA/B and BGTETB/C, which might be hydrolyzed by an unknown endoglucanase, are likely perceived by plasma membrane‐localized OsLecRK1 (Figure 7) to activate in a key step in activating defence signalling during the rice‐BPH interaction. MLG may also function in BPH defence responses in rice in an unknown OsLecRK1‐independent manner. Moreover, natural variants of two alleles in the *OsCslF6* promoter were correlated with MLG‐mediated BPH resistance. Based on biochemical analyses of these two SNPs, we propose that in response to BPH feeding, ARID transcription factors are possibly targeted to the AT‐rich domain in the *OsCslF6* promoter (which is only present in plants carrying the AA allele of SNPs 8 and 9) to enhance OsMYC2‐mediated activation of *OsCslF6* transcription (Figure 7). Thus, our results suggest that *CslF6* genes represent useful targets for molecular breeding to generate broad‐spectrum pest‐resistant cultivars of rice and other cereal crops.

## Materials and methods

### Plant materials and growth conditions

The *Oryza sativa* cultivar Nipponbare (NIP) was used as the wild type and the background for genetic transformation in this study. Fourteen cultivated rice germplasm samples were used for natural variation analysis, including seven with TT alleles and seven with AA alleles (including NIP). The rice seeds were surface sterilized with 75% ethanol for 60 s, followed by 20% NaClO for 30 min. After eight rinses with sterile distilled water, the seeds were germinated on half‐strength Murashige and Skoog (1/2 MS) medium at 28 °C under a short‐day photoperiod (10‐h day/14‐h night) with a light level of 1679 lux. Two‐week‐old seedlings were transferred to a greenhouse under a short‐day photoperiod (10‐h day/14‐h night) with a light level of 21 000 lux at 30 °C.

### Insect materials and BPH feeding assays

The BPH colony used in this study contained mixed biotypes of BPHs and was collected from rice fields in Guangdong. The insects were maintained in wooden cages (50 × 50 × 70 cm) with rice seedlings at 26–28 °C under a 16‐h light/8‐h dark photoperiod. BPH feeding on rice was carried out as described previously (Guo *et al*., 2018). In brief, 4‐week‐old seedlings of different genotypes were infested with BPH. Plants were covered with a light‐transmitting mesh, and 10 BPH third‐instar nymphs per plant were released. BPH honeydew weights were measured as previously described (Chen *et al*., 2010). In brief, a Parafilm sachet was attached to the leaf sheath of each 4‐week‐old seedling, and a female BPH within 1 days after emergence was enclosed in the sachet. After 5 days, the insect was removed and the sachet was weighed.

### Untargeted metabolomic profiling

For untargeted metabolomic profiling, metabolite extraction was performed as previously reported with minor modifications (Kang *et al*., 2019). Four biological replicates were collected for each genotype before and after BPH treatment. Freeze‐dried samples were ground in liquid nitrogen, and each 100 mg sample of powdered plant tissue was extracted with 2 mL of precooled chloroform:methanol mixture (1:2) at 4 °C overnight. After centrifugation, the supernatant was transferred to a new tube, and the extracts were dried under nitrogen gas. The dried samples were resuspended in 200 μL chloroform:methanol mixture (1:2) and filtered prior to LC–MS analysis.

LC–MS analysis was performed on an UPLC‐ESI‐Q‐TOF system (UPLC, Shimadzu Nexera X2; MS, Sciex 5600^+^ Triple TOF). LC was performed using a Dionex Acclaim C18 column (2.6 μm, 2.0 × 150 mm) with a mobile phase consisting of solvent A (ddH_2_O with 0.1% formic acid) and solvent B (acetonitrile with 0.1% formic acid). The gradient program was as follows: 0 min, 5% B; 0–5 min, 5% B–40% B; 5–20 min, 40% B–95% B; 20–22 min, 95% B; 22–25 min, 95% B–5% B; 25–30 min, 5% B. The flow rate was 0.35 mL/min; column temperature 40 °C; injection volume 4 μL. The effluent was connected to the ESI‐QTOF‐MS system in TOF MS + IDA scan mode. The ESI source operation parameters were as follows: ion source, turbo spray; source temperature 550 °C; ion spray voltage (IS) 5500 V (positive ion mode); ion source gas I (GSI), gas II (GSII), and curtain gas (CUR) were set to 55, 55, and 30 psi, respectively. The DP and CE values in TOF MS were set to 100 and 40 V, respectively, while the DP and CE values in IDA were set to 100 and 40 ± 15 V, respectively.

Data analysis was carried out using XCMS online software (Tautenhahn *et al*., 2012; https://xcmsonline.scripps.edu). Feature detection was performed using the centWave method. Retention time correction was performed using the obiwarp method. For alignments, Option was used with the following list parameters: retention time tolerance at 0.5 min, mass tolerance at 5 ppm. Statistical analysis was performed with a paired Welch *t*‐test, and only features whose intensity increased or decreased >1.5‐fold with *P* value < 0.01 were selected as significant features. Annotation and identification of features were based on *m*/*z* within 5 ppm tolerance for the XCMS‐linked database METLIN search.

### Quantitative analysis of MLGs

Endohydrolase‐based measurement of MLG content was performed as previously described (Doblin *et al*., 2009). In brief, alcohol‐insoluble residue (AIR) was prepared by grinding plant material in liquid N_2_ with a mortar and pestle. Each 200‐mg sample was combined with 10 mL of 50% ethanol and incubated in a boiling water bath for 5 min. Following centrifugation at 1800 *g* for 5 min, the supernatant was removed, and the residue was washed twice with 80% ethanol at room temperature, followed by two washes with 100% ethanol for 1 h. The ethanol‐soluble fraction was removed, and the AIR was washed once with 100% ethanol and dried at −20 °C under a vacuum for subsequent experiments. The AIR prepared during the early stage was resuspended in 5 mL NaPO_4_ buffer containing 100 μL β‐(1,3;1,4)‐d‐glucan endohydrolase (lichenase). The mixture was incubated for 15 min at 50 °C with continuous mixing. The supernatant was collected (as the β‐glucan hydrolase‐released oligosaccharides) for HPAEC analysis and for quantitative analyses using a β‐Glucan Assay kit (Megazyme, Dubliin, Ireland).

### HPAEC analysis

The released oligosaccharides were separated by HPAEC on a CarboPacPA1 column (Dionex, Thermo Fisher Scientific, Waltham, MA, USA) equilibrated with 50 mm NaOAc in 0.2 m NaOH using a Dionex BioLC ICS 300 system (Dionex) equipped with a pulsed amperometric detector (PAD, Dionex, Thermo Fisher Scientific, Waltham, MA, USA). Oligosaccharides were eluted at 1 mL/min with a linear gradient of 50 mm NaOAc in 0.2 m NaOH to 350 mm NaOAc in 0.2 m NaOH over a 15 min period. Laminaribiose (Seikagaku, Tokyo, Japan) and cellodextrins (Sigma‐Aldrich, Merck, Darmstadt, Germany) were run as standards.

### ChIP and EMSA

Chromatin immunoprecipitation (ChIP) assays were performed as described (Yuan *et al*., 2017). Briefly, nuclear proteins were extracted from 2‐week‐old *OsMYC2‐OE* transgenic rice leaf sheaths and stems. After coating with anti‐HA (Abiocode, Agoura Hills, CA, USA; HY‐K0201), the protein/DNA complexes were immunoprecipitated with Dynabeads Protein G (Invitrogen, Thermo Fisher Scientific, Waltham, MA, USA) for at least 4 h at 4 °C. The precipitated DNA was purified using a DNA purification kit (Qiagen, Redwood City, CA, USA), and the enriched DNA fragments were subjected to qPCR using specific primers (Table S1). qRT‐PCR was executed as follows: initial denaturation at 95 °C for 5 min, followed by 40 cycles of PCR of denaturing at 95 °C for 10 s and annealing at 60 °C for 30 s. The *OsACTIN1* promoter was used as a negative control.

For the electrophoretic mobility shift assay (EMSA), the amplified *OsMYC2* CDS was fused in‐frame with the His tags in vector pRSET‐A. The recombinant His‐OsMYC2 protein was expressed in *Escherichia coli* BL21 (DE3) cells and purified using Ni‐NTA agarose (Invitrogen). All probes were labelled with biotin using a Biotin 3′ End DNA Labeling Kit (Pierce, Thermo Fisher Scientific, Waltham, MA, USA). Unlabeled oligonucleotides were used as competitors in the binding assay. The oligonucleotide sequences of the probes are listed in Table S1. The EMSA reactions were performed using a LightShift Chemiluminescent EMSA Kit (Pierce) according to the manufacturer's instructions. An appropriate amount of His‐MYC2 protein was incubated in binding buffer (50 ng/μL Poly(dI·dC), 2.5% glycerol, 0.05% NP‐40, 10 mm EDTA, and probes) in a total volume of 20 μL for 30 min at room temperature. After incubation, the binding reactions were loaded onto a 6% polyacrylamide gel and separated by polyacrylamide gel electrophoresis in 0.5× Tris‐Borate‐EDTA buffer at 4 °C. The DNA‐protein complex was transferred to a nylon membrane (Pierce). After cross‐linking, biotin activity was detected according to the manufacturer's instructions of the EMSA kit.

### MST assay

The MST assay was performed as described previously (Zhou *et al*., 2022). In brief, purified MBP‐OsLecRK1‐His and MBP‐OsLecRK2‐His proteins were labelled with reactive dyes using the Monolith RED‐NHS (MO‐L011). The concentration of labelled proteins was adjusted to 10 μm using the labeling buffer NHS buffer (pH 8.2) containing 130 mm NaHCO_3_ and 50 mm NaCl. A serial dilution of various oligosaccharides (BGTRIA, BGTRIB, BGTETB, BGTETC, and CTE) ranging from 1.5 nm to 50 μm was prepared for mixing with the labelled proteins. Next, 90 μL of the prepared protein sample (concentration 10 μm) and 10 μL of the 300 μm dye solution were mixed by gently pipetting several times, then incubated for 30 min in the dark. The mixed samples were filled into standard treated capillaries and fluorescence scanning was performed on a Nano Temper Monolith NT.115 (50% MST power; 30% laser power), to determine binding affinities.

### Phosphorylation assay

The *in vitro* phosphorylation assay was carried out as described by Zhou *et al*. (2022). MBP‐OsLecRK1‐His Recombinant proteins were incubated with the substrate oligosaccharides BGTRIB and BGTETB (10 μm) in reaction buffer (20 mm HEPES pH 7.4, 0.15 m NaCl, 10 mm MgCl_2_ and 3 μL of 10 mm N^6^‐substituted ATPγS) at 25 °C for 15 min. Thereafter, the mixed solution was supplemented with 1.5 μL of 50 mm PNBM at 25 °C for 1 h, followed by the addition of SDS loading buffer for stopping reaction. Finally, phosphorylated MBP‐OsLecRK1‐His protein was detected with a thiophosphate ester specific antibody 51‐8 by immunoblot assay, whereas MBP‐OsLecRK1‐His was detected with anti‐His antibody as a loading control.

### Dual‐luciferase reporter assay

To generate the effector and reporter constructs, the pUC119 and pGreenII‐0800‐LUC vectors were used. All primers used to generate constructs for the transient transactivation assays are listed in Table S1. The transient assays were performed in rice protoplasts via PEG‐mediated transfection, and firefly LUC and Renilla LUC (REN) activities were analysed using the Dual‐Luciferase reporter Assay System (Promega, Madison, Wisconsin, USA) and the SpectraMax i3x Multi‐Mode Detection Platform (Molecular Devices, Shanghai, China) according to the manufacturer's manual. Relative LUC activity is expressed as the ratio of LUC to REN.

### SNP analysis

The 2000‐bp region upstream of *OsCslF6* was retrieved from rice 3K‐sequence web (http://www.rmbreeding.cn/Index/; Wang *et al*., 2020). SNPs in the *OsCslF6* promoters with minor allele frequency (MAF > 0.01) were analysed as described previously (Liu *et al*., 2019).

### Statistical analysis

Statistical analyses throughout the study were carried out using GraphPad 8.3 software. The significance of differences among different groups was determined by one‐way ANOVA with a Tukey's HSD test. The different letters above the figures indicate the statistical significance (*P* < 0.05). The numbers of samples are indicated in the figure legends.

## Conflict of interest

The authors have filed a patent based on the results reported in this study.

## Author contributions

S.X. conceived and designed the experiments. Y.S.D., D.L., W.X.G., Z.X.L., X.Z., L.L.S., D.M.Z., L.N.W., K.K., F.Z.W., S.S.Z., Y.F.T., and T.H. performed experiments. Z.F.Z., Q.M.Z., Z.F.Z., Y.Q.C., and W.Q.Z. contributed to rice materials or field trials. W.C., P.L., and L.Y.Y. collected data. S.X., L.J.Y., J.L. Y.S.D., D.L., and W.X.G. analysed the data and wrote the manuscript.

## Supporting information


**Appendix S1** Additional description of methods.

**Dataset S1** Targeted metabolomic profiling data.

**Dataset S2** Un‐targeted metabolomic profiling data.

**Figure S1** BPH feeding induces the accumulation of jasmonates.
**Figure S2** Generation and identification of *oslox2* and *oscoi1* mutants.
**Figure S3** Jasmonates contribute to resistance to BPH feeding in rice.
**Figure S4** Widely targeted metabolomic profiling showing the differential flavonoid contents in the JA‐related mutants upon BPH feeding.
**Figure S5** Spatial and temporal expression patterns of members of the MLG synthetase *OsCslF* gene family.
**Figure S6** Generation and phenotypic analysis of *OsCslF6* knockout and overexpression transgenic lines.
**Figure S7** *OsMYC2* participates in the plant response to BPH infestation.
**Figure S8** OsMYC2 does not interact with the *OsCslF3*, *OsCslF4*, *OsCslF7*, *OsCslF8*, or *OsCslF9* promoters *in vivo*.
**Figure S9** Overexpression of *OsMYC2* enhances plant resistance to BPH infestation.
**Figure S10** Expression of the *OsARID* gene family upon BPH infestation.
**Figure S11** Generation of *OsLecRK1* and *OsLecRK3* double knockout lines.
**Table S1** Primers used in this study.
