# Supplementary material for: Poaceae‐specific β‐1,3;1,4‐d‐glucans link jasmonate signalling to OsLecRK1‐mediated defence response during rice‐brown planthopper interactions
Source: Plant Biotechnol J. 2023 Mar 23;21(6):1286–300. doi: 10.1111/pbi.14038 (PMC10214751; doi:10.1111/pbi.14038)
Supplement: Supplementary file 1 — Appendix S1 Additional description of methods. [file PBI-21-1286-s003.docx]

**Supplementary methods**

**RNA extraction and gene expression analysis**

Total RNA was extracted from the samples with TRIzol reagent (Invitrogen) and reverse transcribed using a PrimeScript RT reagent kit with gDNA Eraser (Takara). Quantitative PCR analysis was performed using the StepOne Plus Real-time PCR system (Applied Biosystems) with SYBR Premix ExTaqMix (Takara). qRT-PCR analysis was performed as follows: initial denaturation at 95°C for 5 min, followed by 40 cycles of denaturing at 95°C for 10 sec, and annealing at 60°C for 30 sec. *OsACTIN1* was used as the reference gene. Three technical replicates were performed per reaction. Semi-quantitative RT-PCR analysis was carried out as follows: initial denaturation at 95°C for 3 min, followed by 30 cycles (*OsMYC2*) or 24 cycles (*OsACTIN1*) of denaturing at 95°C for 30 sec, and annealing at 55°C for 30 sec, and extension at 72°C for 30 sec. *OsACTIN1* was used as an internal control. The gene-specific primers used for qRT-PCR and semi-quantitative RT-PCR analysis are listed in Table S1.

**Phytohormone measurements**

Phytohormones were extracted from the samples as previously described (Yuan *et al*., 2017). Briefly, 200-mg fresh leaf sheath and stem tissues were collected for total hormone extraction. The samples were dissolved in 100 μL of a solution of 60 μL methanol and 40 μL distilled water. A 10-µL aliquot of each sample was injected into a C18 column and analyzed with the AB Sciex Q-TOF 5600^+^ system as described (Chen *et al*., 2015).

**Generation of transgenic rice plants**

To generate knockout mutants by CRISPR/Cas9, 20 bp of conserved sequence in *OsLOX2*, *OsCOI1a*, *OsCOI1b*, *OsCslF6*, *OsLecRK1* and *OsLecRK3* regions was selected as the target site. The sequences were ligated to the pYLCRISPR/gRNA vector, followed by ligation to the pYLCRISPR/Cas9-MTmono vector after dual-nested PCR as previously described (Ma *et al*., 2015). The target site sequences were amplified with specific primer pairs using PCR, followed by sequencing and alignment with the target site sequences to confirm the mutated sites.

For the *oslox2*-*cas9* lines, the target sequence (CAGACGCCCAAGGGGATCG) was selected before the Protospacer Adjacent Motif (PAM) (AGG). Sequencing analysis showed that the *oslox2-1* line had two inserted bases (T and G) at the third and sixth nucleotides in front of the PAM respectively, leading to a frameshift of OsLOX2 protein from the 226^th^ amino acid and premature termination at the 230^th^ amino acid. By contrast, the *oslox2-2* line had one inserted base (G) at the sixth nucleotide in front of the PAM, leading to a frameshift of *OsLOX2* from the 226^th^ amino acid.

For *oscoi1a/1b-cas9* double mutants, the target sequence (ATGGGGCCCTGGAGTCTATT) before the PAM (GGG), a region with same base sequence in both *OsCOI1a* and *OsCOI1b* genomic sequences, was selected. Sequencing analysis showed that the *oscoi1-1* line contains two deleted bases (TC) at the fourth and fifth positions in front of the PAM in *OsCOI1a* and one deleted base (C) at the fifth positions in front of the PAM in *OsCOI1b*, leading to a frameshift of OsCOI1a protein from the 435^th^ amino acid and premature termination at the 451^st^ amino acid, and a frameshift of OsCOI1b protein from the 402^th^ amino acid and premature termination at the 424^th^ amino acid. Moreover, the *oscoi1-2* line contains one deleted base (C) at the fifth position in front of the PAM in *OsCOI1a* and two deleted bases (TC) at the fourth and fifth positions the fifth positions in front of the PAM in *OsCOI1b*, leading to a frameshift of OsCOI1a protein from the 435^th^ amino acid and a premature terminator at the 457^th^ amino acid, and a frameshift of OsCOI1b protein from the 402^th^ amino acid and premature termination at the 419^th^ amino acid.

For the *oslf6*-*cas9* lines, the target sequence (GGGAGGAGGAACAATG) was selected before the PAM (AGG). The sequencing results showed that the *f6cas3* line had four deleted bases (G and CAA) at the first and third-to-fifth sites in front of the PAM respectively, leading to a frameshift of OsCsLF6 protein from the 14^th^ amino acid and a premature terminator at the 16^th^ amino acid. By contrast, the *f6cas10* line had one deleted base (G) at the first site in front of the PAM, leading to a frameshift of OsCslF6 from the 15^th^ amino acid and a premature terminator at the 17^th^ amino acid.

For the *oslecrk1/3-cas9* line, the target sequence of *OsLecRK1* (CATGAAGAGGCAGGACTCTGG) and *OsLecRK3* (GTCGTCTGGTACGCAAGG) was selected before the PAM (TGG) and the PAM (AGG), respectively. Sequencing analysis showed that the *lecrk1/3* line had six deleted bases (CTCTGG) at the first three bases containing the PAM (TGG), leading to a frameshift of OsLecRK1 protein from the 30^th^ amino acid and premature termination at the 49^th^ amino acid, and had four deleted bases (GGTA) at the fifth to eighth sites in front of the PAM (AGG), leading to a frameshift of OsLecRK3 protein from the 79^th^ amino acid and premature termination at the 140^th^ amino acid.

For *F6OE4 lecrk1/3* line, the same sequence of targets and PAMs of *OsLecRK1* and *OsLecRK3* as *oslecrk1/3-cas9* line were selected and edited by CRISPR-cas9 system in the *F6OE4* genotype background. Sequence analysis showed that the *F6OE4 lecrk1/3* line had four deleted bases (AGGA) at the fourth to seventh sites in front of the PAM (TGG), leading to a frameshift of OsLecRK1 protein from the 35^th^ amino acid and premature termination at the 53^th^ amino acid, and had one inserted base (T) at the third to fourth sites in front of the PAM (AGG), leading to a frameshift of OsLecRK3 protein from the 81^th^ amino acid and premature termination at the 84^th^ amino acid.

To generate overexpression plants, the entire cDNA sequence of *OsMYC2* and *OsCslF6* was amplified using specific primer pairs (Table S1) and cloned into the pFGC-RCS binary vector driven by the *UBQ10* promoter (Qi *et al*., 2017).

The *OsMYC2* RNAi lines were generated by PCR amplification of a 0.5-kb sense *OsMYC2* coding sequence (CDS) fragment using the primer pair *OsMYC2*-RNAi-S-F and *OsMYC2*-RNAi-S-R (Table S1) and its corresponding 0.5-kb antisense fragment using the primer pair *OsMYC2*-RNAi-AS-F and *OsMYC2*-RNAi-AS-R (Table S1). The sense and antisense fragments were respectively cloned into the *Xho*I-*Eco*RI and *Hind*III-*Xba*I sites of binary vector pFGC-pHANNIBAL (which was modified from pHANNIBAL) (Xiao *et al*., 2010). The sequence-confirmed plasmids were introduced into *A. tumefaciens* strain EHA105, followed by transformation into rice cultivar NIP.

**Targeted metabolomic profiling**

Widely targeted metabolomic analysis was performed by Metware (Wuhan, China) using UPLC-ESI-MS/MS (UPLC, Shimadzu Nexera X2; MS, Sciex 4500 Q TRAP). Three biological replicates were collected for each genotype before and after BPH treatment. Freeze-dried rice leaf sheath and stem samples were crushed with a mixer mill, and a 100 mg sample of lyophilized powder was extracted with 70% methanol at 4°C overnight. After centrifugation, the extracts were filtered prior to UPLC-MS/MS analysis (Chen *et al*., 2013).

LC analysis was performed using an Agilent SB-C18 column with a mobile phase consisting of solvent A (ddH_2_O with 0.1% formic acid) and solvent B (acetonitrile with 0.1% formic acid). The gradient program was as follows: 0 min, 5% B; 0–9 min, 5% B–95% B; 9–10 min, 95% B; 10–11.1 min, 95% B–5% B; 11.1–14 min, 5% B. The flow rate was 0.35 mL/min; column temperature was 40°C; injection volume was 4 μL. The effluent was connected to an ESI-QTRAP-MS system. The ESI source operation parameters were as follows: ion source, turbo spray; source temperature 550°C; ion spray voltage (IS) 5500 V (positive ion mode)/-4500 V (negative ion mode); ion source gas I (GSI), gas II (GSII), curtain gas (CUR) was set at 50, 60, and 25.0 psi, respectively. QQQ scans were acquired as MRM experiments with collision gas (nitrogen) set to medium. DP and CE were optimized for individual MRM transitions. A specific set of MRM transitions was monitored for each period according to the metabolites eluted within that period. Significantly differentially abundant metabolites between groups were determined by VIP ≥ 1 and absolute log_2_(fold change) ≥ 1. VIP (Variable importance in the projection) scores were designated as the values that extracted from the OPLS-DA results, which also contained score plots and permutation plots, as generated using the R package MetaboAnalystR. The data were log transformed (log_2_) and subjected to mean centering prior to OPLS-DA.

**Protein extraction and immunoblot analysis**

Rice protoplast cells were isolated from 1-week-old leaf sheaths by digesting with cellulase (Cellulase RS, [Yakult](https://www.rjmart.cn/v/#/detail?productId=200007285442&suppId=68556)) for 4 h, followed by transfecting for 12 h. Protoplast cells were harvested by centrifuging at 800 *g* for 5 min and 200 μl protein extraction buffer (50 mM Tris-HCl, pH 7.5, 150 mM NaCl, 0.5% [v/v] Triton X-100) containing protease inhibitor cocktail (Roche Basel, Switzerland; 04693132001) were added for total protein extraction.

For immunoblot analysis, extracted proteins were separated using SDS-PAGE and then transferred to a Hybond-C membrane (Amersham Biosciences, Amersham, UK). Photos of immunoblotting using different antibodies was detected by a CDD camera, and quantification of protein accumulation was performed by Image J software. Antibodies against the following proteins were used as follows: HA (Sigma-Aldrich; cat. No. H6533, 1:5,000), Thiophosphate ester (Abcam; ab92570, 1:5,000), and Plant ACTIN (Abbkine, Wuhan, China; cat. No. A01050, 1:5,000).

**Transmission electron microscopy**

Transmission electron microscopy was performed as previously described with minor revision (Guo et al., 2018). Briefly, fresh leaf sheaths were fixed in 0.1 M PBS (pH 7.2) with 5% glutaraldehyde and 4% paraformaldehyde using a vacuum pump for air extraction, until the samples were completely sunk into the fixation solution. The samples were subsequently washed in PBS, refixed with 2% osmium tetroxide overnight, dehydrated in a concentration gradient of ethanol and finally embedded in epoxy resin. For transmission electron microscopy, the samples were sectioned into ultrathin sections using an ultramicrotome machine (Leica EM UC7). Before observation, these sections were stained with uranyl acetate for 15 min, followed by staining with lead citrate for 15 min. Finally, observations of these section were performed with a transmission electron microscope system (JEOL Ltd. JEM-1400). For vascular bundle observation, the epoxy resin-embedded samples were sectioned into semithin sections (10 μm) and stained with toluidine blue. Then, the vascular bundle cells were observed with a confocal microscopy system (Leica TCS SP8 X).
