# Supplementary material for: Poaceae‐specific β‐1,3;1,4‐d‐glucans link jasmonate signalling to OsLecRK1‐mediated defence response during rice‐brown planthopper interactions
Source: Plant Biotechnol J. 2023 Mar 23;21(6):1286–300. doi: 10.1111/pbi.14038 (PMC10214751; doi:10.1111/pbi.14038)
Supplement: Supplementary file 4 — Figure S1 BPH feeding induces the accumulation of jasmonates. Figure S2 Generation and identification of oslox2 and oscoi1 mutants. Figure S3 Jasmonates contribute to resistance to BPH feeding in rice. Figure S4 Widely targeted metabolomic profiling showing the differential flavonoid contents in the JA‐related mutants upon BPH feeding. Figure S5 Spatial and temporal expression patterns of members of the MLG synthetase OsCslF gene family. Figure S6 Generation and phenotypic analysis of OsCslF6 knockout and overexpression transgenic lines. Figure S7 OsMYC2 participates in the plant response to BPH infestation. Figure S8 OsMYC2 does not interact with the OsCslF3, OsCslF4, OsCslF7, OsCslF8, or OsCslF9 promoters in vivo. Figure S9 Overexpression of OsMYC2 enhances plant resistance to BPH infestation. Figure S10 Expression of the OsARID gene family upon BPH infestation. Figure S11 Generation of OsLecRK1 and OsLecRK3 double knockout lines. Table S1 Primers used in this study. [file PBI-21-1286-s001.docx]

**Supporting Information**

**Supporting Figures**


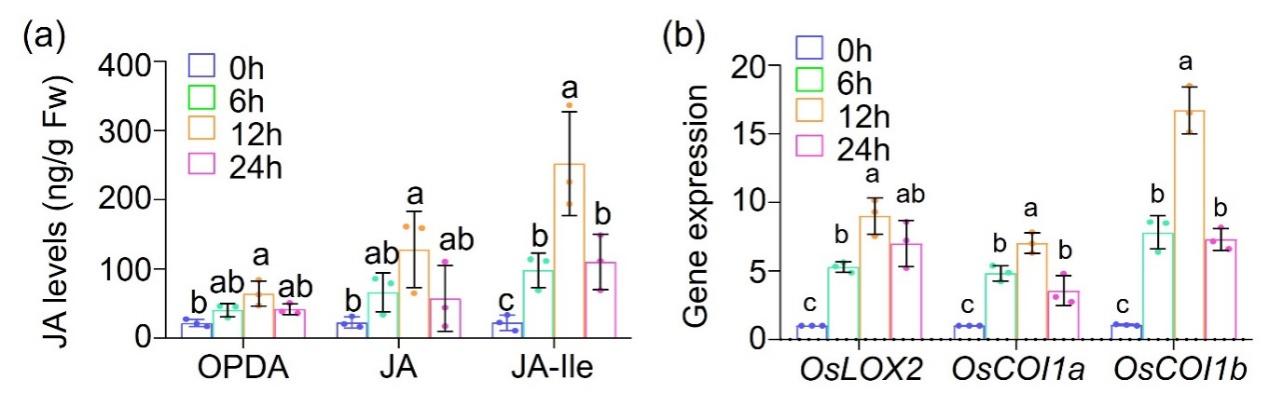


**Supplemental Figure 1. BPH feeding induces the accumulation of jasmonates.**

(a) Endogenous jasmonate (OPDA, JA, and JA-Ile) levels in rice leaf sheaths and stems in response to BPH feeding. The leaf sheaths and stems of 4-week-old NIP plants at 0, 6, 12, and 24 h after BPH infestation were collected for jasmonate extraction and subjected to LC-MS analysis. H_2_-JA was added as an internal quantitative standard. Data are means ± SD (*n* = 3 biological replicates). Fw, Fresh weight. (b) RT-qPCR analysis showing the BPH-inducible expression of the jasmonate biosynthetic gene *OsLOX2* and jasmonate signaling genes *OsCOI1a* and *OsCOI1b*. Total RNA was extracted from the leaf sheaths and stems of 4-week-old NIP plants at 0, 6, 12, and 24 h of BPH feeding. Transcript levels relative to 0 h (prior to treatment) for each time point were normalized to the level of *OsACTIN1*. Data are means ± SD (*n* = 3 biological replicates). Letters indicate significant differences between groups conducted by one-way ANOVA, *P* < 0.05.


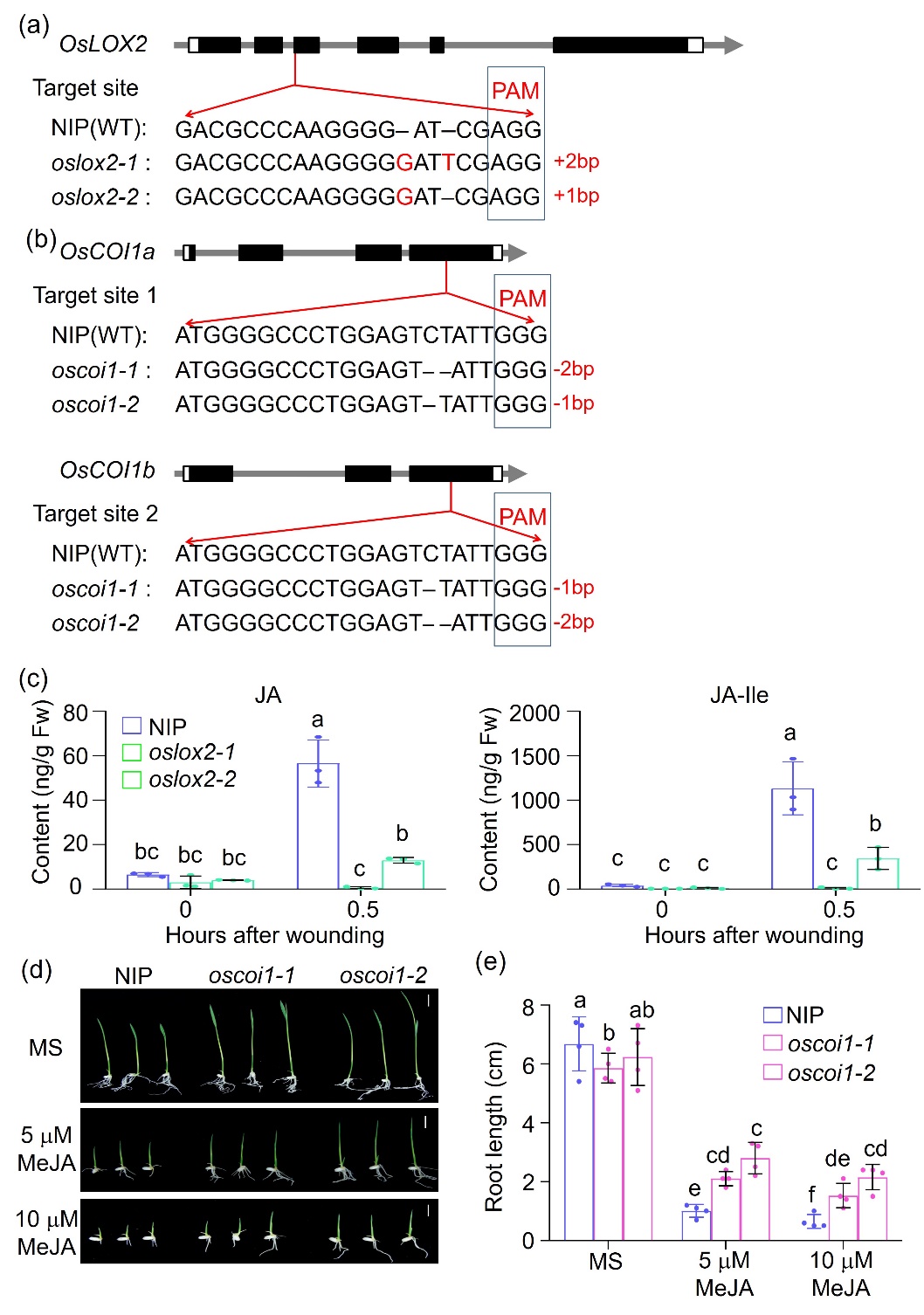


**Supplemental Figure 2. Generation and identification of *oslox2* and *oscoi1* mutants.**

(a) Gene structure of *OsLOX2* and sequence alignment of the *oslox2* mutants produced with CRISPR-Cas9. The *oslox2* mutants included a 2-bp insertion in *oslox2-1* and a 1-bp insertion in *oslox2-2* at the target site. PAM, Protospacer Adjacent Motif. (b) Gene structures of *OsCOI1a* and *OsCOI1b*, and sequence alignments of the *oscoi1* mutants produced with CRISPR-Cas9. The *oscoil1-1* mutant contained a 2-bp deletion in *OsCOI1a* and a 1-bp deletion in *OsCOI1b*; the *oscoil1-2* mutant had a 1-bp deletion in *OsCOI1a* and a 2-bp deletion in *OsCOI1b*. (c) Endogenous jasmonate (JA and JA-Ile) levels in NIP and *oslox2* leaf sheaths and stems in response to wounding treatment. The leaf sheaths and stems of 4-week-old NIP, *oslox2-1*, and *oslox2-2* plants at 0 and 0.5 h after wounding treatment were used for jasmonate extraction and LC-MS analysis. H_2_-JA was added as an internal quantitative standard. Data are means ± SD (*n* = 3 biological replicates). (d) Phenotypes of germinated NIP, *oscoi1-1*, and *oscoi1-2* seeds grown on MS or MS medium containing 5 and 10 μM MeJA for 7 d. Scale bars, 1 cm. (e) Root elongation in the NIP, *oscoi1-1*, and *oscoi1-2* seedlings shown in (d). Root lengths were measured at 7 d after germination. Data are means ± SD (*n* = 4 biological replicates; 10 roots were measured for each replicate). Letters indicate significant differences between groups conducted by one-way ANOVA, *P* < 0.05.


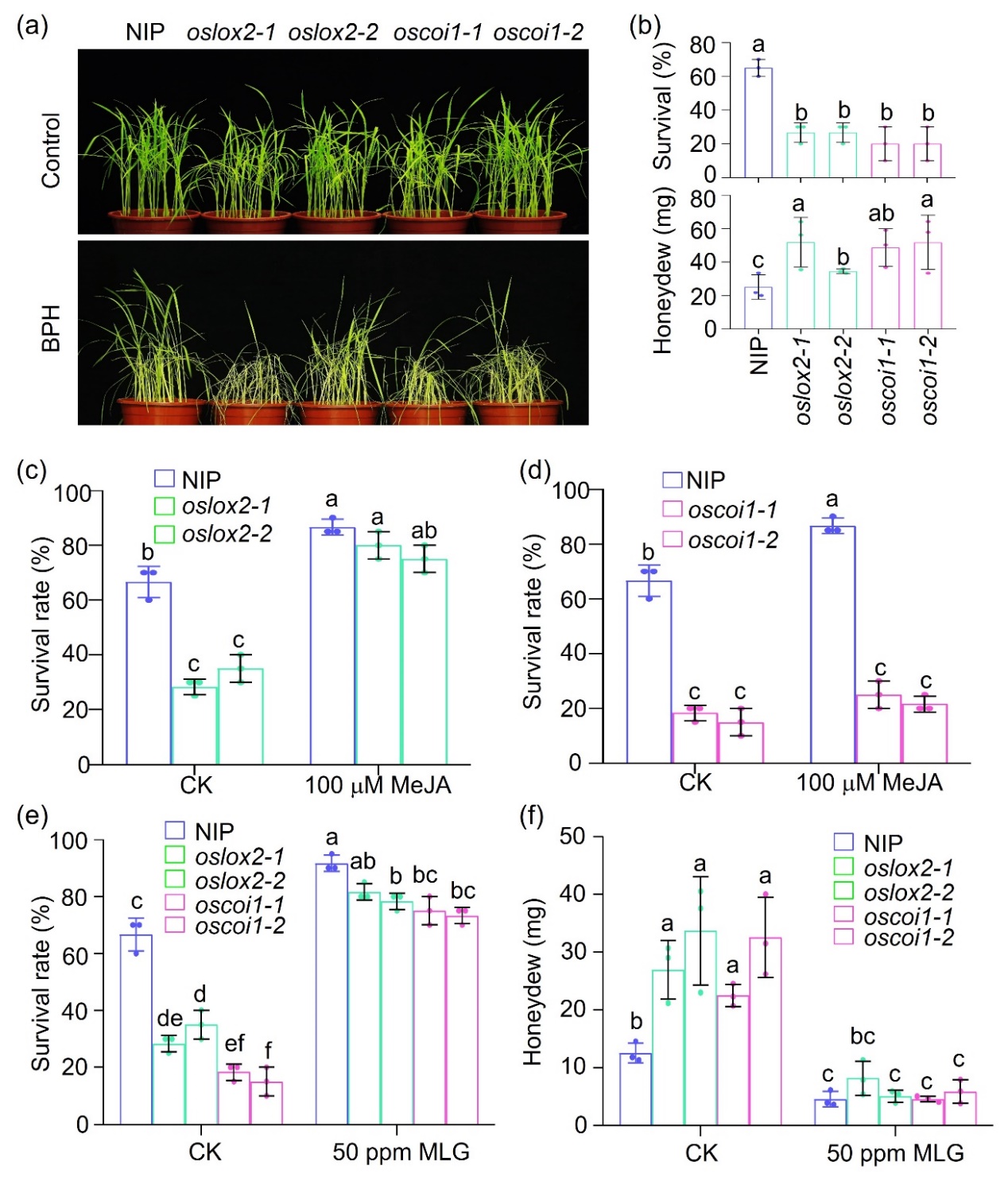


**Supplemental Figure 3. Jasmonates contribute to resistance to BPH feeding in rice.**

(a) Phenotypes of 4-week-old NIP, *oslox2* (*oslox2-1* and *oslox2-2*), and *oscoi1a/1b* double mutant (*oscoi1-1* and *oscoi1-2*) plants infested with BPH for 5 d. (b) Survival rates and honeydew weights of plants infested with BPH in (a). The survival rate and honeydew weights were determined after BPH feeding for 5 d. (c and d) Survival rates of NIP, *oslox2-1*, *oslox2-2* (c), *oscoi1-1*, and *oscoi1-2* (d) plants infested with BPH upon pre-treatment without (CK; water) and 100 mM MeJA for 24 h. The survival rates were determined after MeJA treatment and BPH infestation for 5 d. (e and f) Survival rates (e) and honeydew weights (f) of NIP, *oslox2-1*, *oslox2-2*, *oscoi1-1*, and *oscoi1-2* plants infested with BPH upon pre-treatment without (CK, water) or with 50 ppm MLGs for 24 h. The survival rates and honeydew weights were determined after BPH with MLG treatment for 5 d. Data are means ± SD (*n* = 3 biological replicates; for each replicate, 10 and 30 plants per genotype were calculated for survival rates and honeydew weights, respectively). Letters indicate significant differences between groups conducted by one-way ANOVA, *P* < 0.05.


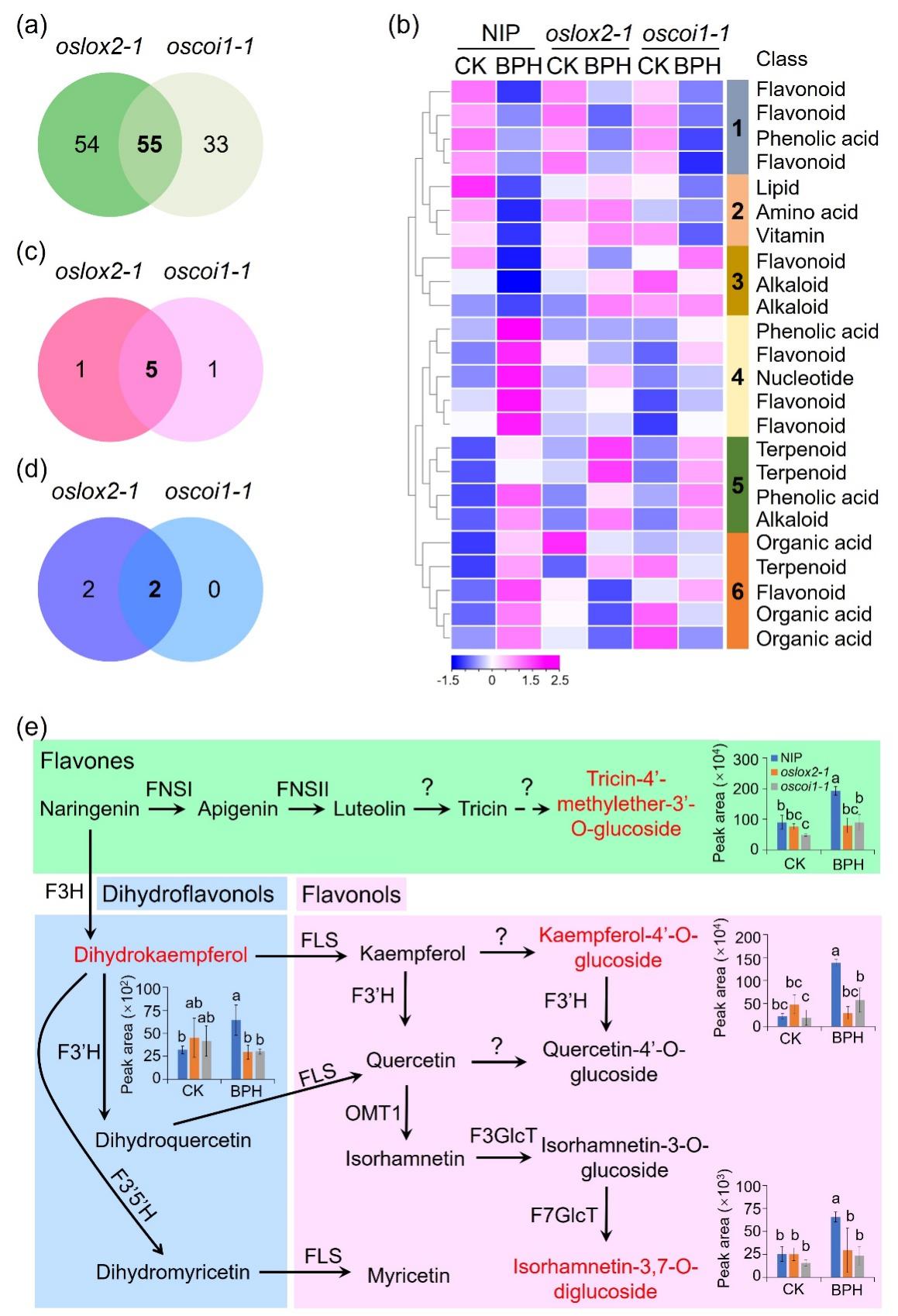


**Supplemental Figure 4. Widely targeted metabolomic profiling showing the differential flavonoid contents in the JA-related mutants upon BPH feeding.**

(a-d) Venn diagrams and heatmap of metabolite levels in plants under BPH treatment. Leaf sheaths and stems of 4-week-old NIP, *oslox2-1*, and *oscoi1-1* plants at 0 (CK) and 1 d (BPH) after infestation with BPH were collected and subjected to widely targeted metabolomics analysis. (a) Diagram showing different features (VIP ≥ 1 and |Fold change| ≥ 2) in each group of NIP vs. *oslox2-1* (109) or NIP vs. *oscoi1-1* (88). (b) Widely targeted metabolomic profiling showing potential jasmonate-regulated metabolites that were significantly different in 4-week-old NIP leaf sheaths and stems upon BPH infestation (VIP ≥ 1 and |Fold change| ≥ 2). Standard-scores (Z-scores) were used to indicate the means of arbitrary peak abundance units (*n* = 3 biological replicates) of the corresponding samples. (c) Diagram showing features that accumulated to significantly higher levels in NIP plants compared to *oslox2-1* (6) and *oscoi1-1* (6) plants upon BPH infestation (VIP ≥ 1 and |Fold change| ≥ 2). (d) Diagram showing features whose levels decreased upon BPH infestation in NIP plants but accumulated in *oslox2-1* (4) or *oscoi1-1* (2) plants (VIP ≥ 1 and |Fold change| ≥ 2). VIP, Variable importance in the projection. (e) Simple flavonoid biosynthesis pathway map showing the position of potential jasmonate-regulated flavonoids, including one flavone (tricin-4′-methyl ether-3′-*O*-glucoside), two flavonols (kaempferol-4′-*O*-glucoside and isorhamnetin-3,7-*O*-diglucoside) and one dihydroflavonol (dihydrokaempferol), which were induced in NIP plants upon BPH infestation compared with NIP plants without BPH feeding, while these compounds were reduced in jasmonate mutants (*oslox2-1* and *oscoi1-1*) upon BPH infestation compared with NIP plants upon BPH infestation. Column diagram showing the peak area of these four flavonoids that were detected by the widely target metabolomics analysis. Data are means ± SD (*n* = 3 biological replicates). Letters indicate significant differences between groups conducted by one-way ANOVA analysis, *P* < 0.05.


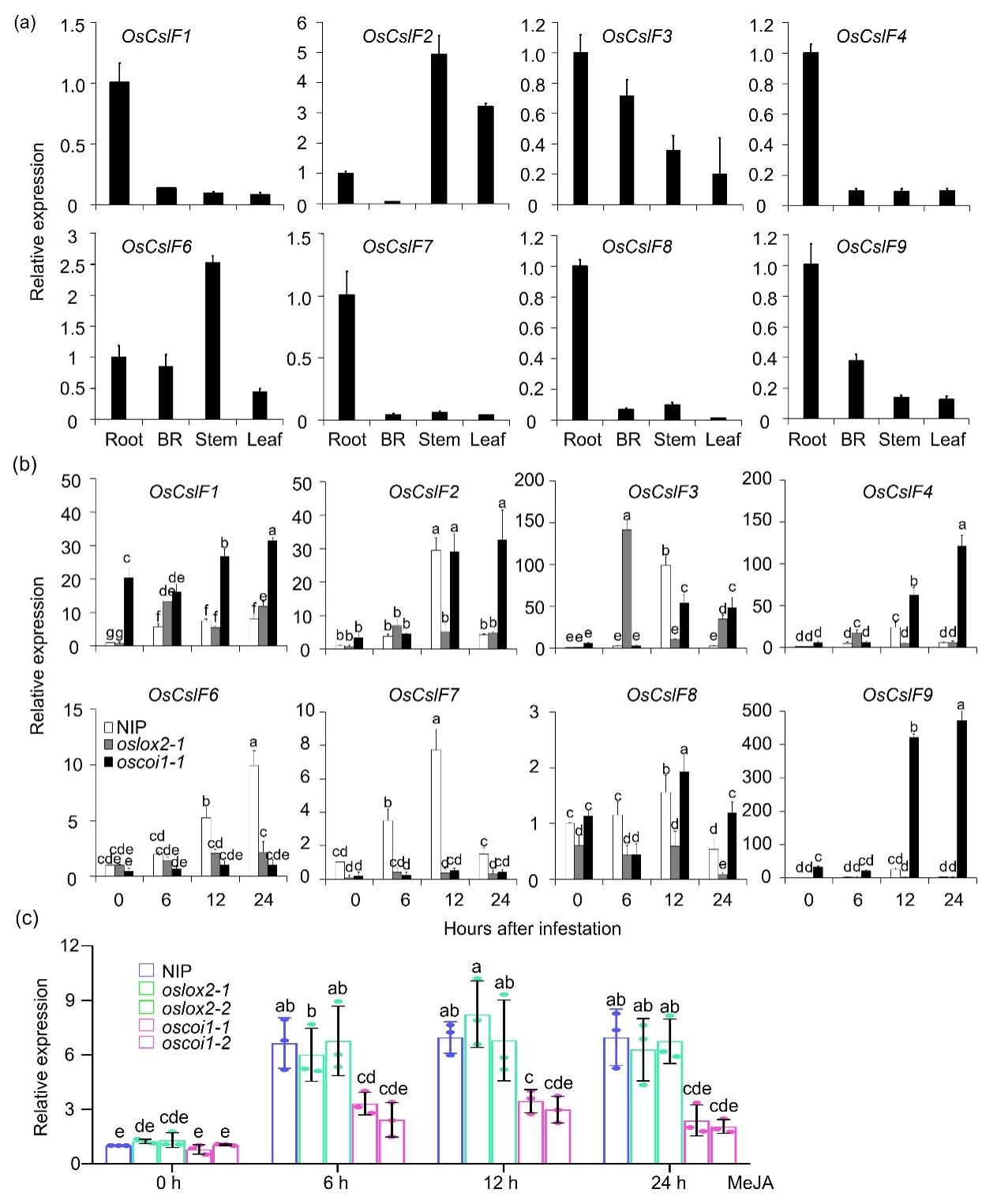


**Supplemental Figure 5. Spatial and temporal expression patterns of members of the MLG synthetase *OsCslF* gene family.**

(a) RT-qPCR analysis showing the expression of *OsCslF* genes in the root, BR (basal regions), stem (including leaf sheath), and leaf of 4-week-old wild-type NIP. Transcript levels in different tissues relative to roots were normalized to the levels of *OsACTIN1*. (b) RT-qPCR analysis showing the BPH-inducible expression levels of *OsCslF* genes in NIP, *oslox2-1*, and *oscoi1-1*. Total RNA was extracted from the leaf sheaths and stems of 4-week-old plants at 0, 6, 12, and 24 h of BPH feeding. Transcript levels relative to 0 h (prior to treatment) for each time point were normalized to the levels of *OsACTIN1*. Data are means ± SD (*n* = 3 biological replicates). Letters indicate significant differences between groups conducted by one-way ANOVA, *P* < 0.05. (c) RT-qPCR analysis showing the MeJA-inducible expression of *OsCslF6* in NIP, *oslox2* (*oslox2-1* and *oslox2-2*), and *oscoi1a/1b* double mutant (*oscoi1-1* and *oscoi1-2*) plants. Total RNA was extracted from the leaf sheaths and stems of 2-week-old plants at 0, 6, 12, and 24 h after treatment with 100 μM MeJA. Transcript levels relative to NIP at 0 h for each time point in each genotype were normalized to the level of *OsACTIN1*. Data are means ± SD (*n* = 3 biological replicates). Letters indicate significant differences between groups conducted by one-way ANOVA, *P* < 0.05.

**
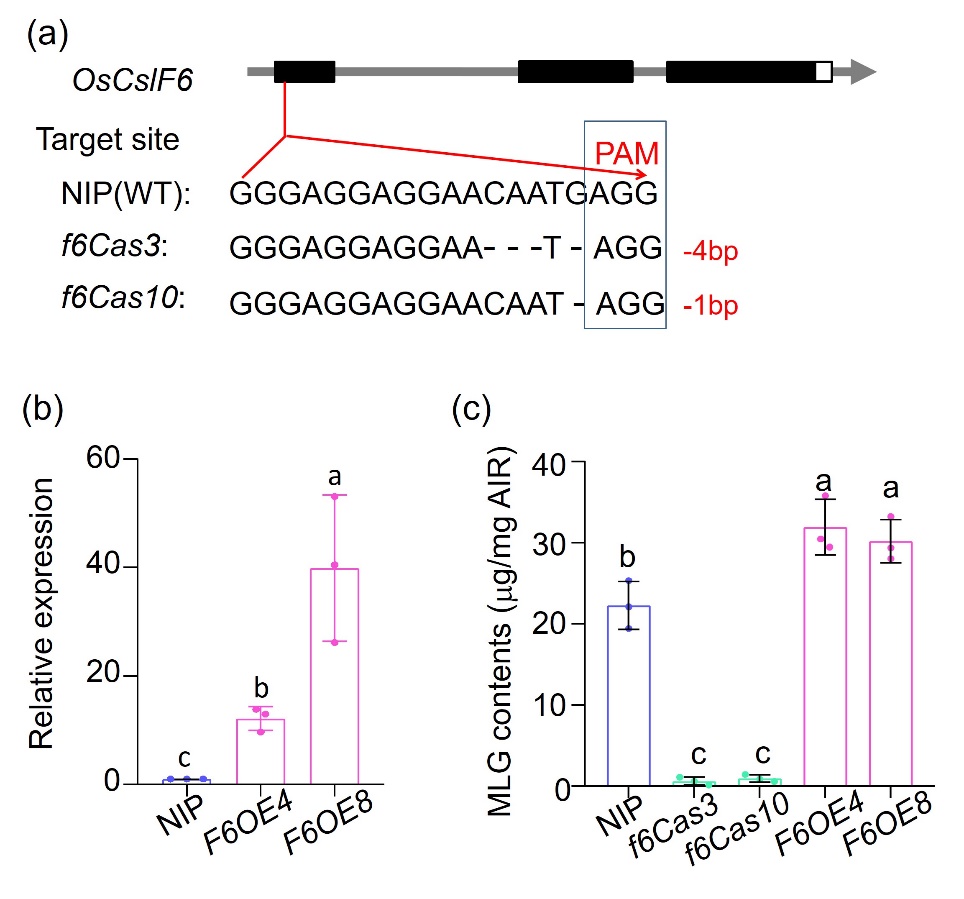
**

**Supplemental Figure 6. Generation and phenotypic analysis of *OsCslF6* knockout and overexpression transgenic lines.**

(a) Gene structure of *OsCslF6* and sequence alignment of the *oscslf6* mutants constructed with CRISPR-Cas9. The *oscslf6* mutants included a 4-bp deletion in *f6Cas3* and a 1-bp deletion in *f6Cas10* at the target site. (b) RT-qPCR analysis showing that *OsCslF6* was expressed at higher levels in *OsCslF6* overexpressing (*F6OE4* and *F6OE8*) lines vs. NIP plants. Total RNA was extracted from the leaf sheaths and stems of 4-week-old NIP, *F6OE4*, and *F6OE8* plants. Transcript levels relative to NIP for each genotype were normalized to the levels of *OsACTIN1*. Data are means ± SD (*n* = 3 biological replicates). (c) MLG contents in 4-week-old NIP, *f6Cas3*, *f6Cas10*, *F6OE4*, and *F6OE8* plants infested with BPH. Data are means ± SD (*n* = 3 biological replicates). Letters indicate significant differences between groups conducted by one-way ANOVA, *P* < 0.05.


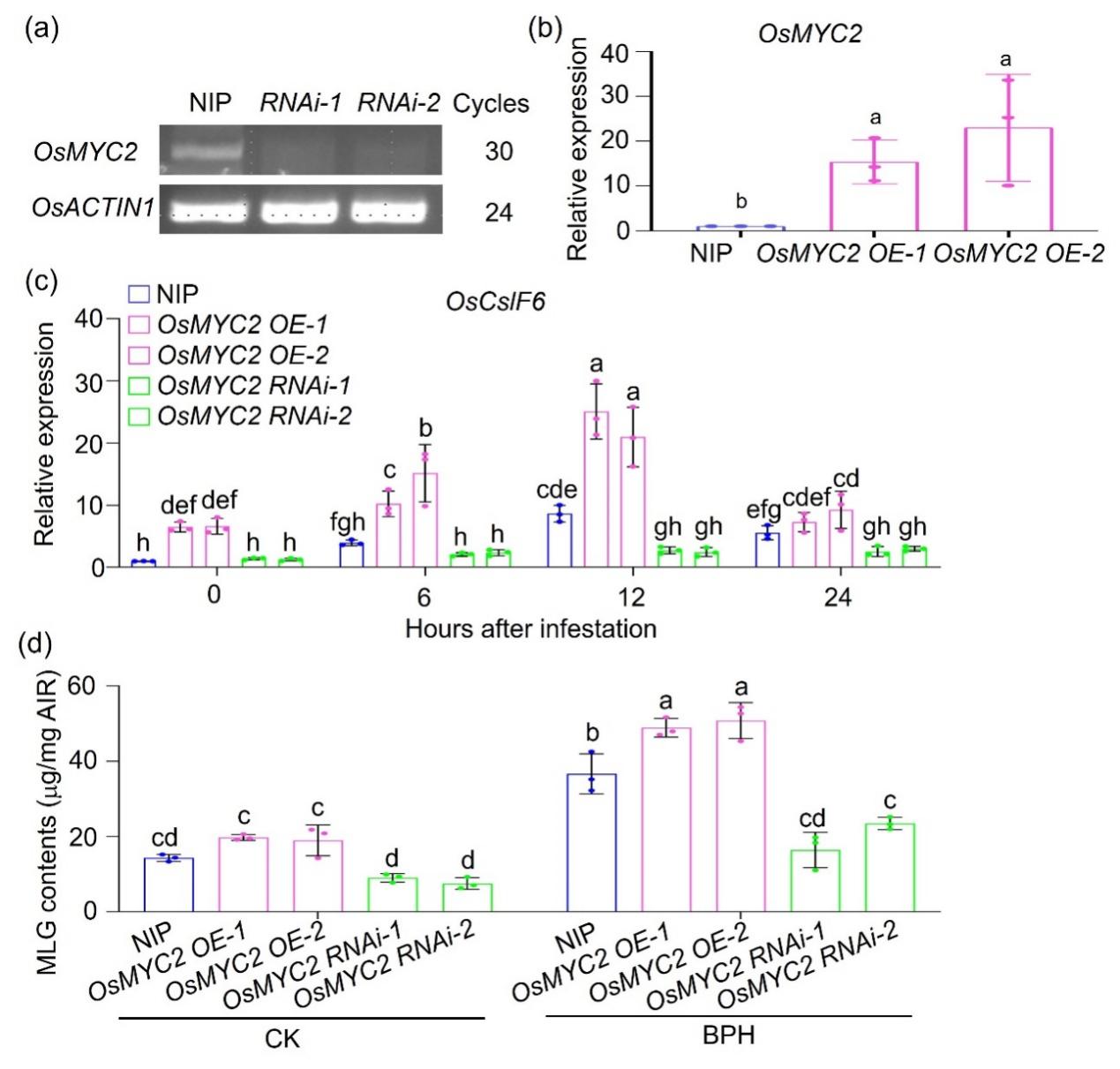


**Supplemental Figure 7. *OsMYC2* participates in the plant response to BPH infestation.**

(a) Semi-quantitative RT-PCR analysis showing that *OsMYC2* was expressed at lower levels in *OsMYC2-RNAi* transgenic lines (*RNAi-1* and *RNAi-2*) vs. NIP plants. Total RNA was extracted from the leaf sheaths and stems of 4-week-old NIP, *RNAi-1*, and *RNAi-2* plants. *OsACTIN1* was used as an internal control. (b) RT-qPCR analysis showing that *OsMYC2* was expressed at higher levels in *OsMYC2-*overexpressing lines (*OsMYC2 OE-1* and *OsMYC2 OE-2*) vs. NIP plants. Total RNA was extracted from the leaf sheaths and stems of 4-week-old NIP, *OsMYC2 OE-1*, and *OsMYC2 OE-2* plants. Transcript levels relative to NIP for each genotype were normalized to the level of *OsACTIN1*. Data are means ± SD (*n* = 3 biological replicates). (c) RT-qPCR analysis showing the BPH-inducible expression of *OsCslF6* in *OsMYC2-*overexpressing (*OsMYC2 OE-1* and *OsMYC2 OE-2*) and OsMYC2-RNAi (*OsMYC2* *RNAi-1* and *OsMYC2* *RNAi-2*) plants. Total RNA was extracted from the leaf sheaths and stems of 4-week-old NIP, *OsMYC2 OE-1*, *OsMYC2 OE-2*, *OsMYC2* *RNAi-1* and *OsMYC2* *RNAi-2* plants at 0, 6, 12, and 24 h upon BPH feeding. Transcript levels relative to NIP at 0 h for each time point in each genotype were normalized to the level of *OsACTIN1*. Data are means ± SD (*n* = 3 biological replicates). (d) MLG contents in the leaf sheaths and stems of 4-week-old NIP, *OsMYC2 OE-1*, *OsMYC2 OE-2*, *OsMYC2* *RNAi-1* and *OsMYC2* *RNAi-2* plants upon BPH infestation for 0 (CK) and 1 d (BPH). Data are means ± SD (*n* = 3 biological replicates). Letters indicate significant differences between groups conducted by one-way ANOVA, *P* < 0.05.


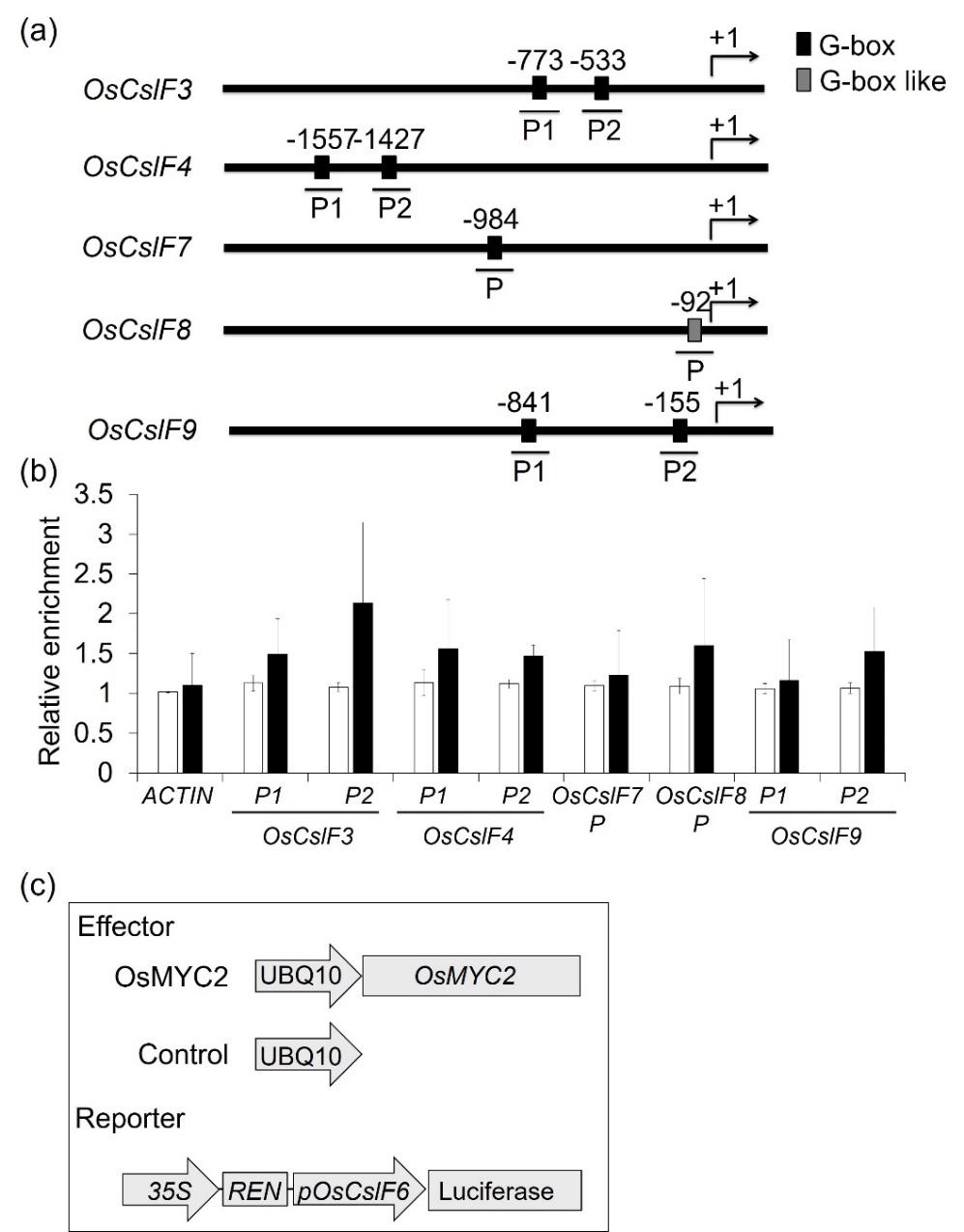


**Supplemental Figure 8. OsMYC2 does not interact with the *OsCslF3*, *OsCslF4*, *OsCslF7*, *OsCslF8*, or *OsCslF9* promoters *in vivo*.**

(a) Schematic diagram of the potential G-box and G-box-like motifs in the promoter fragments of *OsCslF3*, *OsCslF4*, *OsCslF7*, *OsCslF8*, and *OsCslF9*. Numbers indicate the nucleotide positions relative to their translational start sites, which are shown as +1. (b) ChIP-qPCR analysis showing the *in vivo* interaction of OsMYC2 with DNA fragments in the promoters of *OsCslF3*, *OsCslF4*, *OsCslF7*, *OsCslF8*, and *OsCslF9.* Protein-DNA complexes isolated from 2-week-old *OsMYC2-OE* plants were amplified from immunoprecipitated proteins pulled down with or without anti-HA antibodies. For each promoter, the enrichment of DNA fragments containing the G-box and G-box-like motifs was determined. The *OsACTIN1* promoter fragment was used as a negative control. Data are means ± SD (*n* = 3 biological replicates). (c) Effector and reporter constructs used in the transient LUC assays. Construct consisting of the *UBQ10* promoter driving *OsMYC2* or empty vector were used as effectors. The dual-luciferase reporter construct consisted of the 35S promoter driving the Renilla luciferase (REN) reporter gene (expressed for normalization) and the promoters of *pOsCslF6* driving the reporter gene encoding firefly LUC.


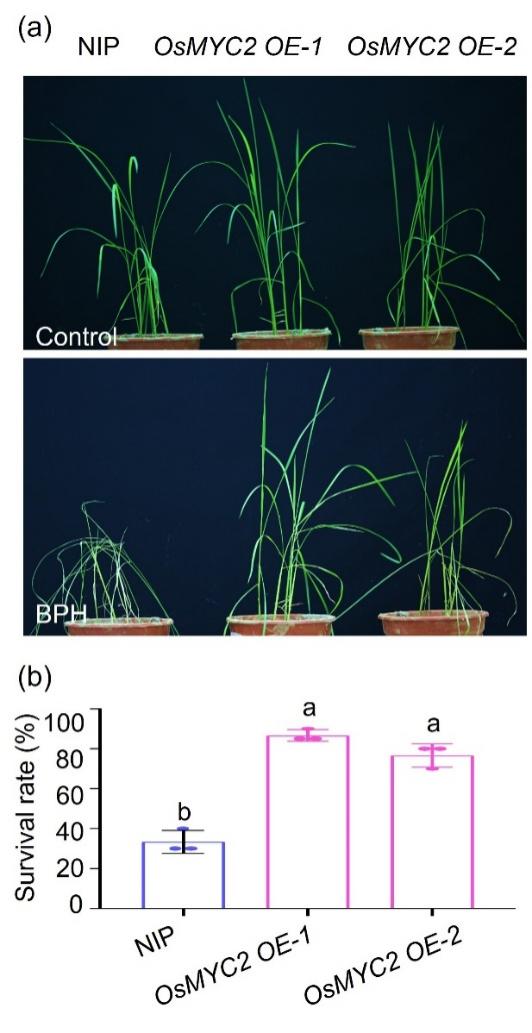


**Supplemental Figure 9. Overexpression of *OsMYC2* enhances plant resistance to BPH infestation.**

(a) Phenotypes of NIP and *OsMYC2-*overexpressing (*OsMYC2 OE-1* and *OsMYC2 OE-2*) plants infested with BPH for 7 d. (b) Survival rates of various genotypes infested with BPH (a). The survival rates were determined after treatment with BPH for 7 d. Data are means ± SD (*n* = 3 biological replicates). For each replicate, 10 plants per genotype were used for the calculation. Letters indicate significant differences between groups conducted by one-way ANOVA, *P* < 0.05.


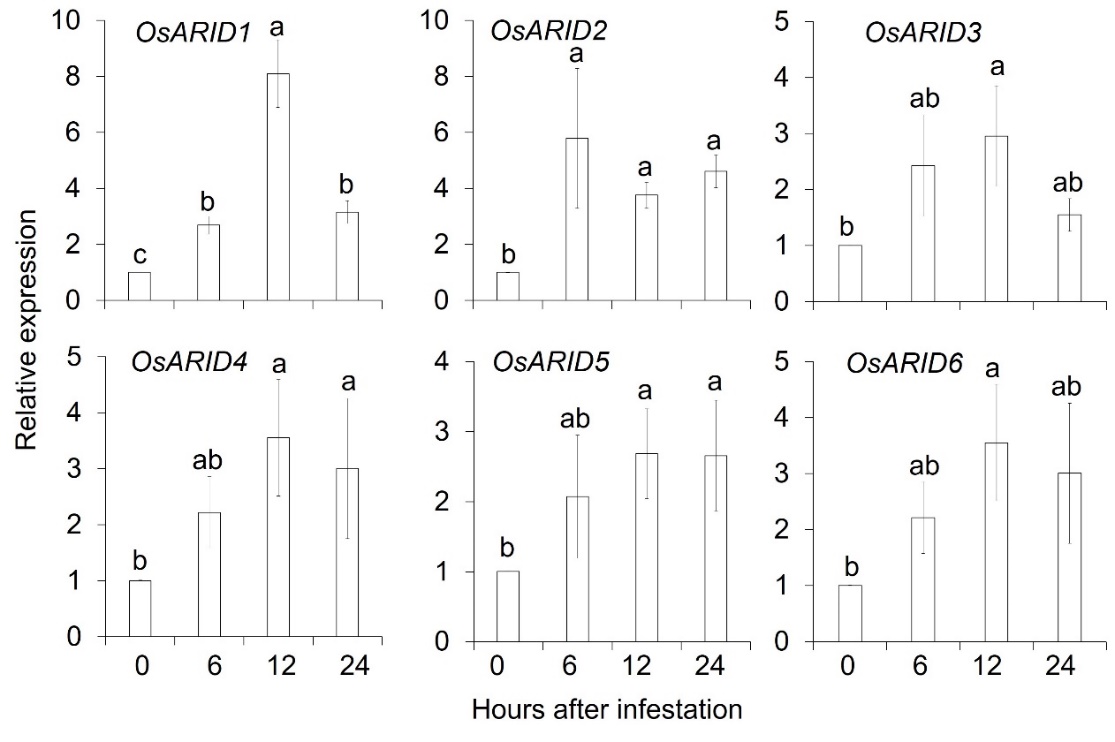


**Supplemental Figure 10. Expression of the *OsARID* gene family upon BPH infestation.**

RT-qPCR analysis showing the BPH-inducible expression levels of *OsARID* genes in NIP plants. Total RNA was extracted from the leaf sheaths and stems of 4-week-old plants at 0, 6, 12, and 24 h upon BPH feeding. Transcript levels relative to 0 h (prior to treatment) for each time point were normalized to the levels of *OsACTIN1*. Data are means ± SD (*n* = 3 biological replicates). Letters indicate significant differences between groups conducted by one-way ANOVA, *P* < 0.05.


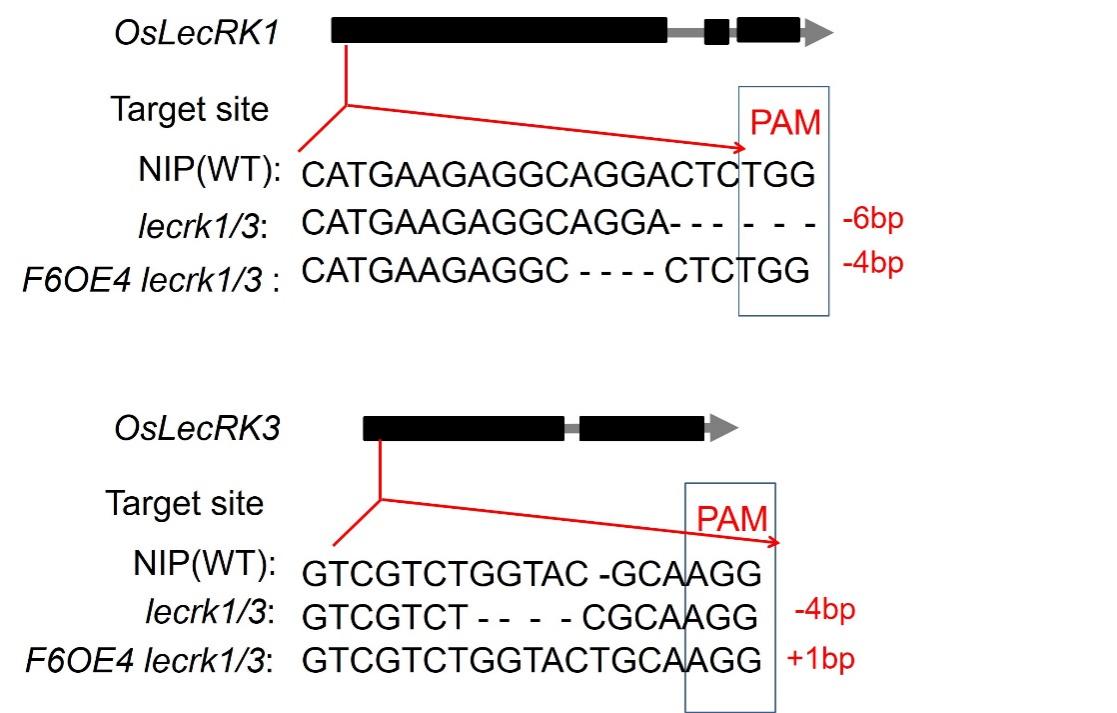


**Supplemental Figure 11. Generation of *OsLecRK1* and *OsLecRK3* double knockout lines.**

Gene structure of *OsLecRK1* and *OsLecRK3* and sequence alignment of the *oslecrk1/3* mutants constructed with CRISPR-Cas9. The *lecrk1/3* mutants have a 6-bp deletion in *OsLecRK1* and a 4-bp deletion in *OsLecRK3* at the target site. The *F6OE4 lecrk1/3* mutants have a 4-bp deletion in *OsLecRK1* and a 1-bp insertion in *OsLecRK3* at the target site. PAM, Protospacer Adjacent Motif.

**Table S1 Primers used in this study.**

| **Primer Name** | **Sequence (5′–3′)** |  |
| --- | --- | --- |
| **Primers for transgenic plant construction** | |  |
| *oslox2*-cas9-tag-F | GTTGCAGACGCCCAAGGGGATCG |  |
| *oslox2*-cas9-tag-R | AAACCGATCCCCTTGGGCGTCTG |  |
| *oscoi1a/1b*-cas9-tag-F | GGCAATGGGGCCCTGGAGTCTAT |  |
| *oscoi1a/1b*-cas9-tag-R | AAACATAGACTCCAGGGCCCCAT |  |
| *oscslf6*-cas9-tag-F | CTTGTAGGGGAGGAGGAACAATG |  |
| *oscslf6*-cas9-tag-R | AAACCATTGTTCCTCCTCCCCTA |  |
| *OsCslF6*-OE-F | CGACTCTAGCGGATCCATGGCGCCAGCGG |  |
| *OsCslF6*-OE-R | GTCCTTGTAGTCAGGCCTTGGCCAGGCGTAGGTG |  |
| *OsMYC2*-OE-F | TGATTAACAGGGATCCATGTGGGTTTTGTTATCTCCTCTCCT |  |
| *OsMYC2*-OE-R  *OsLecRK1*-OE-F  *OsLecRK1*-OE-R | GTCGTATGGGTAAGGCCTCCGGGCGGCGGT  TGATTAACAGGGATCATGGTTGCTCTGCTACTC  GTCGTATGGGTAAGGTGGAAGTGAGCTGATGAAGG |  |
| *OsMYC2*-RNAi-S-F | CACGCTCGAGGAATTCCAACCTCAGCGCCGCC |  |
| *OsMYC2*-RNAi-S-R | TTGGGGTACCGAATTCTGCGAGAACAGGCTCCC |  |
| *OsMYC2*-RNAi-AS-F | CGAAATCGATAAGCTTTGCGAGAACAGGCTCCCC |  |
| *OsMYC2*-RNAi-AS-R | TAGAGGATCCAAGCTTCAACCTCAGCGCCGC |  |
| **Primers for identification of transgenic plants** | |  |
| *oslox2*-cas9-F | GGACGATGAAGCAGGACACG |  |
| *oslox2*-cas9-R | GAGGTGAGGTGGGGTTACCG |  |
| *oscoi1a*-cas9-F | CAAGAAGCTACGAAGACTCCGA |  |
| *oscoi1a*-cas9-R | GTAGCACAAGCCGAAAGTCGT |  |
| *oscoi1b*-cas9-F | AGACTCAGAGTTGAGCGAGGA |  |
| *oscoi1b*-cas9-R | AGGACAAGACGGAAGTCGCA |  |
| *oscslf6*-cas9-F | CCTCCTCTTGTGTGGAGCTC |  |
| *oscslf6*-cas9-R  *oscslecrk1*-cas9-F  *oscslecrk1*-cas9-R  *oscslecrk3*-cas9-F  *oscslecrk3*-cas9-R | GGTTCACACTCTTGCAACTCTTCC  AGCAAGAGCCTGAAGCAAAGA  CATCAAGGAGAAACCGACCAC  TTGCCCATCCTGCAACTCTT  ACCCACCATTGTTTGTATTGACTG |  |
| *OsMYC2*-ID-Q-F | TGGACGTGTACCATGCCAG |  |
| *OsMYC2*-ID-Q-R | AGCTGGTCCTGCGAGTAGAC |  |
| *OsMYC2*-RNAi-ID-F | GAACCTTTGGACGGACGACA |  |
| *OsMYC2*-RNAi-ID-R | CGGAGCTCGTTGATGTAGGA |  |
| **Primers for RT-qPCR** | |  |
| Q-*OsACTIN1*-F | CATTGGTGCTGAGCGTTTCC |  |
| Q-*OsACTIN1*-R | CCCGCAGCTTCCATTCCTAT |  |
| Q-*OsLOX2*-F | GCGAGATAGGTTCTCGTGGT |  |
| Q-*OsLOX2*-R | TGCTCACGATGGGAAACTCC |  |
| Q-*OsCOI1a*-F | GATGCCCTCCCTGAGATACA |  |
| Q-*OsCOI1a*-R | AGTCAGACCTCCTTCCAGCA |  |
| Q-*OsCOI1b*-F | TCTGGGTAATGTTGGGGAAA |  |
| Q-*OsCOI1b*-R | GCCCTGTACCCACACGTATC |  |
| Q-*OsCsLF1*-F | GTGATCGGCATGTTCGAGGT |  |
| Q-*OsCsLF1*-R | GATGCCGCCGCCACTTTAC |  |
| Q-*OsCsLF2*-F | CAGGTTCCTGATCCTTCTTCG |  |
| Q-*OsCsLF2*-R | AGCCTCGGTTTACAGCTTGG |  |
| Q-*OsCsLF3*-F | CGCATACGAGGATGGGACG |  |
| Q-*OsCsLF3*-R | TCGACGTGAGCCGGAAGTAGA |  |
| Q-*OsCsLF4*-F | AACAGAGCGAAGGATGGCAA |  |
| Q-*OsCsLF4*-R | TTGACGTAGTGGTCGCAGTC |  |
| Q-*OsCsLF6*-F | TTCTGGTTCGGGTTCTCGTG |  |
| Q-*OsCsLF6*-R | TAGGTGAGGAGCATCCCAGA |  |
| Q-*OsCsLF7*-F | CTCGGGCTGATGGGTCGC |  |
| Q-*OsCsLF7*-R | GTAACAAGAGACAAAGGAAGCGGA |  |
| Q-*OsCsLF8*-F | TGGTCTACCAGCAAGCACTG |  |
| Q-*OsCsLF8*-R | GAAGGAGTCCAAGTTGCCGA |  |
| Q-*OsCsLF9*-F | TCCACATCCAGAAGCCGTTC |  |
| Q-*OsCsLF9*-R | GCTTAAGTCGCCAGCTTGAC |  |
| Q-*OsARID1*-F | CTTCACGAGGTGGCATTGAT |  |
| Q- *OsARID1*-R | AATCTGCAGTTTCTTGATACCAGC |  |
| Q-*OsARID2*-F | CTGCCGAGTGGGTGAAGATT |  |
| Q- *OsARID2*-R | TGGAGTGATCCCCCAAGGAT |  |
| Q-*OsARID3*-F | CGGCAAGTGGGAGAGTCTTT |  |
| Q- *OsARID3*-R | TTCACCATTGCCAAGCAAGC |  |
| Q-*OsARID4*-F | TGTTAAGCTTGGGCCTCGTT |  |
| Q- *OsARID4*-R | AGCATTCAAACCATCCTCCAGT |  |
| Q-*OsARID5*-F | ACCGGTGTTGGAAACACACT |  |
| Q- *OsARID5*-R | GCCCATTCACCACACAAACC |  |
| Q-*OsARID6*-F | TTCCATTCCCTCATGGGCAC |  |
| Q- *OsARID6*-R | GCAAGTCCATCTCCTTGCCT |  |
| **Primers for ChIP-qPCR** | |  |
| ChIP-*OsACTIN1*-F | ATAAACCCTATCACTATGTCTGGTGTTTC |  |
| ChIP-*OsACTIN1*-R | CCATTGTTGACATAGAGGCTAGGTTT |  |
| ChIP-*OsCslF3*-P1-F | GCTACTTCTGCTTAACTGCAAC |  |
| ChIP-*OsCslF3*-P1-R | GCCGCCTTAATTACGGCCTG |  |
| ChIP-*OsCslF3*-P2-F | GTACAGAGCATGCATGCAAAGC |  |
| ChIP-*OsCslF3*-P2-R | CCACTGGTGAACAAAACGTGC |  |
| ChIP-*OsCslF4*-P1-F | CGATGCTCTCCTTGACCTCTCT |  |
| ChIP-*OsCslF4*-P1-R | GCCGGGTTTTGTATGAGAAGAC |  |
| ChIP-*OsCslF4*-P2-F | CATCATCAGGAGTTCAGAACTC |  |
| ChIP-*OsCslF4*-P2-R | CCGAACCGAGTACGTACTCAAG |  |
| ChIP-*OsCslF6*-P1-F | GGCTGTCATAACTGCATTGC |  |
| ChIP-*OsCslF6*-P1-R | CTAGTTACAAACCCCTTTCTGTG |  |
| ChIP-*OsCslF6*-P2-F | TATATTGTTGGCTAGCTAGCTAAG |  |
| ChIP-*OsCslF6*-P2-R | GAGCATGAGAGGCCCATATG |  |
| ChIP-*OsCslF7*-F | CTCTCTCTCCTGAAGTATATTTAC |  |
| ChIP-*OsCslF7*-R | GTCCTAACTCGTATAATGTG |  |
| ChIP-*OsCslF8*-F | CCAGCGTCCAAACTCCAAATC |  |
| ChIP-*OsCslF8*-R | GGAATAGTGCATTAGGTTTTAG |  |
| ChIP-*OsCslF9*-P1-F | GACGGGCTGCAAGTACACCA |  |
| ChIP-*OsCslF9*-P1-R | GATGTAACTCAAACTCAGGTC |  |
| ChIP-*OsCslF9*-P2-F | GCAGCTAGCCCACTCCGG |  |
| ChIP*-OsCslF9-P2-R* | GTACGTGTGCGCGAGTGTG |  |
| ChIP-*SNP89*-P1-F | TATATTGTTGGCTAGCTAGCTAAG |  |
| ChIP-*SNP89*-P1-R | GAGCATGAGAGGCCCATATG |  |
| ChIP-*SNP89*-P2-F | CTAGCTAGCTAAGTTTTGTGCC |  |
| ChIP-*SNP89*-P2-R | GAGCATGAGAGGCCCATATG |  |
| ChIP-*SNP89*-P3-F | CCTCTTCCTCTTCCCTGATTAAAC |  |
| ChIP-*SNP89*-P3-R | GCTAGCTAGCCAACAATATAATTTGG |  |
| **Primers for EMSA** | |  |
| *OsCslF6*-P1-F | TTGAAGAAGAAGAAACACATCACACATGCAGCAAGCGCTGCAAAGCAAGG | |
| *OsCslF6*-P1-R | CCTTGCTTTGCAGCGCTTGCTGCATGTGTGATGTGTTTCTTCTTCTTCAA |  |
| *OsCslF6*-P1-mF | TTGAAGAAGAAGAAACACATCATTTTTTCAGCAAGCGCTGCAAAGCAAGG |  |
| *OsCslF6*-P1-mR | CCTTGCTTTGCAGCGCTTGCTGAAAAAATGATGTGTTTCTTCTTCTTCAA |  |
| *OsCslF6*-P2-F | CTAGCTAAGTTTTGTGCCATATCACATGGCAAGAGGAGGAATATGAGTGG |  |
| *OsCslF6*-P2-R | CCACTCATATTCCTCCTCTTGCCATGTGATATGGCACAAAACTTAGCTAG |  |
| *OsCslF6*-P2-mF | CTAGCTAAGTTTTGTGCCATATTTTTTTGCAAGAGGAGGAATATGAGTGG |  |
| *OsCslF6*-P2-mR | CCACTCATATTCCTCCTCTTGCAAAAAAATATGGCACAAAACTTAGCTAG |  |
| His-OsMYC2-F | GGATCGATGGGGATCCATGTGGGTTTTGTTATCTCCTCTCC |  |
| His-OsMYC2-R | TCTCGAGCTCGGATCCTTACCGGGCGGCGGTGCC |  |
| **Primers for dual-luciferase reporter assay** | |  |
| UBQ10-OsMYC2-F | TGATTAACAGGGATCCATGTGGGTTTTGTTATCTCCTCTCCT |  |
| UBQ10-OsMYC2-R | GTCGTATGGGTAAGGCCTCCGGGCGGCGGT |  |
| *pOsCslF6-LUC*-F | TATAGGGCGAATTGGGTACCGCTTTCCTCCTCTTGTTTTAAGCATATGTG |  |
| *pOsCslF6-LUC*-R | TAGAACTAGTGGATCCTGCTAATGCCTTTGCCTCTCCC |  |
| **Primers for purified protein vector construction** | |  |
| MBP-*OsLecRK1*-His-F | TATCGTCGACGGATCCATGGTTGCTCTGCTACTC |  |
| MBP-*OsLecRK1*-His-R | GATGATGATGGGATCCTGGAAGTGAGCTGATGAAGG |  |
| MBP-*OsLecRK2*-His-F | TATCGTCGACGGATCCATGGCACCTCTCCTGT |  |
| MBP-*OsLecRK2*-His-R | GATGATGATGGGATCCTGCGAGTGAACTGATATAGGACG |  |
